# Distinct myeloid antigen-presenting cells dictate differential fates of tumor-specific CD8^+^ T cells in pancreatic cancer

**DOI:** 10.1172/jci.insight.151593

**Published:** 2022-04-08

**Authors:** Adam L. Burrack, Zoe C. Schmiechen, Michael T. Patterson, Ebony A. Miller, Ellen J. Spartz, Meagan R. Rollins, Jackson F. Raynor, Jason S. Mitchell, Tsuneyasu Kaisho, Brian T. Fife, Ingunn M. Stromnes

**Affiliations:** 1 Department of Microbiology and Immunology,; 2 Center for Immunology,; 3 Department of Medicine, and; 4 Department of Laboratory Medicine and Pathology, University of Minnesota Medical School, Minneapolis, Minnesota, USA.; 5 Department of Immunology, Institute of Advanced Medicine, Wakayama Medical University, Kimiidera, Wakayama, Japan.; 6 Masonic Cancer Center, and; 7 Center for Genome Engineering, University of Minnesota Medical School, Minneapolis, Minnesota, USA.

**Keywords:** Immunology, Antigen-presenting cells, Cancer immunotherapy, T cells

## Abstract

We investigate how myeloid subsets differentially shape immunity to pancreatic ductal adenocarcinoma (PDA). We show that tumor antigenicity sculpts myeloid cell composition and functionality. Antigenicity promotes accumulation of type 1 dendritic cells (cDC1), which is driven by Xcr1 signaling, and overcomes macrophage-mediated suppression. The therapeutic activity of adoptive T cell therapy or programmed cell death ligand 1 blockade required cDC1s, which sustained splenic Klrg1^+^ cytotoxic antitumor T cells and functional intratumoral T cells. *KLRG1* and cDC1 genes correlated in human tumors, and PDA patients with high intratumoral *KLRG1* survived longer than patients with low intratumoral *KLRG1*. The immunotherapy CD40 agonist also required host cDC1s for maximal therapeutic benefit. However, CD40 agonist exhibited partial therapeutic benefit in cDC1-deficient hosts and resulted in priming of tumor-specific yet atypical CD8^+^ T cells with a regulatory phenotype and that failed to participate in tumor control. Monocyte/macrophage depletion using clodronate liposomes abrogated T cell priming yet enhanced the antitumor activity of CD40 agonist in cDC1-deficient hosts via engagement of innate immunity. In sum, our study supports that cDC1s are essential for sustaining effective antitumor T cells and supports differential roles for cDC1s and monocytes/macrophages in instructing T cell fate and immunotherapy response.

## Graphical abstract

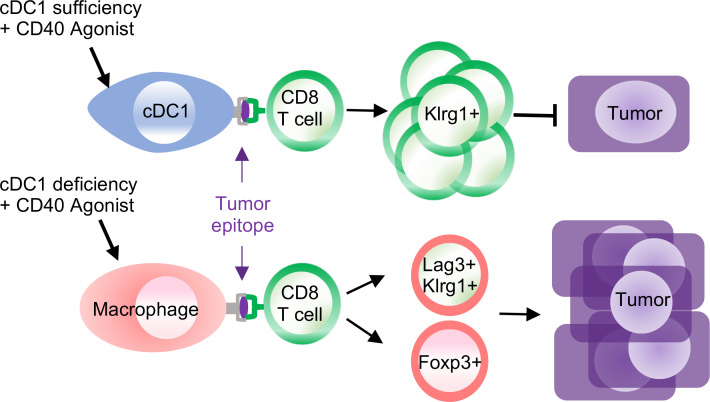

## Introduction

The incidence of pancreatic ductal adenocarcinoma (PDA) is increasing and is predicted to become the second leading cause of cancer deaths in the United States by 2030 (1). Late diagnosis, propensity for metastasis, and resistance to cytotoxic therapies contribute to disease lethality. PDA is often refractory to immune checkpoint blockade (2–4), which is transforming the standard of care for several other advanced solid tumors (5). Recently, a phase I clinical trial using a combination of a CD40 agonist and standard-of-care chemotherapy (nab-paclitaxel + gemcitabine), with or without anti–programmed cell death 1 (αPD-1) elicited clinical responses in a subset of evaluable patients with metastatic PDA, suggesting that immune-based treatments can target advanced disease (6). Complications of this regimen included 2 deaths, both attributed to chemotherapy-induced sepsis and potentially resistance. Thus, a major effort is underway to understand the mechanistic basis for immunotherapy antitumor activity to inform safe and efficacious treatments for patients with PDA.

PDA orchestrates a suppressive tumor microenvironment (TME) particularly enriched for protumor myeloid cells, including tumor-associated macrophages (TAMs) and myeloid-derived suppressor cells (MDSCs) (7, 8), while interfering with prototypical antitumor myeloid subsets, such as type 1 conventional dendritic cells (cDC1s) (9–11). In situ analysis of human PDA shows rare DCs localized in tertiary lymphoid structures, whereas TAMs surround tumor cells and colocalize with CD8^+^ T cells in the stroma (7). The underlying factors that pancreatic tumor cells produce, including M-CSF, GM-CSF, and G-CSF, promote suppressive myeloid cell accumulation while interfering with cDC1 development (9–14).

cDC1s require the transcription factor *Batf3* for development, express Xcr1, and are critical for antitumor immunity (15–18). cDC1s are highly efficient at crosspresentation of cell-associated antigen to prime naive CD8^+^ T cells (19). Activated CD8^+^ T cells produce Xcl-1 (20) to promote cDC1 interactions (21). cDC1s are licensed by CD4^+^ T cells, can activate naive CD4^+^ T cells (22), and produce IL-12, which instructs antitumor T cell differentiation (23). cDC1s are critical for the activation of autoreactive T cells that cause type 1 diabetes (24) and for the accumulation of adoptively transferred T cells in murine melanoma (25). Therapies that promote cDC1s enhance immune checkpoint blockade in preclinical cancer models (9, 10). The impact of cDC1s is often assessed using *Batf3^–/–^* mice, but Batf3 can function in non-cDC1 immune cells (26–29). Thus, alternative approaches to specifically target cDC1s have been developed (30) and employed here.

The genetically engineered *Kras^G12D/+^ Trp53^R172H/+^ p48-Cre* (*KPC*) PDA mouse model recapitulates many of the hallmark features of human PDA (31). However, this model may not fully recapitulate the mutational burden and spectrum of T cell infiltrate in human tumors and lacks defined antigens for tumor-specific T cell analysis. Among total immune cells, roughly half of human PDAs contain 30%–40% CD8^+^ T cells (7, 32), whereas the proportion of CD8^+^ T cells in *KPC* tumors ranges from 1% to 10% (32, 33). The T cell–infiltrated subset among human PDAs has also been transcriptionally identified (34). *KPC* tumor cell lines that express the standard model antigen, ovalbumin (Ova), are rejected or result in Ova loss variants in vivo (35, 36). Therefore, we generated *KPC* tumor cells that express click beetle red luciferase (CB) (36, 37), which is typically used for in vivo imaging (38). However, CB also contains an immunogenic epitope, which elicits the expansion of endogenous tumor-specific CD8^+^ T cells detected via a fluorescent CB_101-109_:H-2D^b^ tetramer (36, 37). Following orthotopic tumor implantation, CB-specific T cells accumulate within tumors but are rapidly rendered exhausted and fail to control tumor growth or elicit antigen loss (36, 37). CB^+^ *KPC* orthotopic tumors are resistant to monotherapy with anti–programed cell death ligand 1 (αPD-L1) or αPD-1 (36) and respond to agonistic αCD40 + αPD-L1 (37), resembling results of clinical trials in patients with PDA (6, 39). As most PDAs have mutations in *KRAS* and *TRP53* (40), which can produce neoepitopes among various HLAs (41–43), the translational relevance of the CB^+^ PDA model may extend beyond microsatellite instability–high tumors (44, 45). We use this model to identify the role of myeloid subsets on tumor-specific T cells, tumor progression, and immunotherapy response. Together, our results highlight the role of tumor antigenicity in sculpting intratumoral myeloid cells and identify both cDC1-dependent and -independent mechanisms governing T cell fate and tumor control.

## Results

### Protumor roles for TAMs and granulocytic myeloid-derived suppressor cells are obviated in neoantigen^+^ PDA.

**Figure 1:**
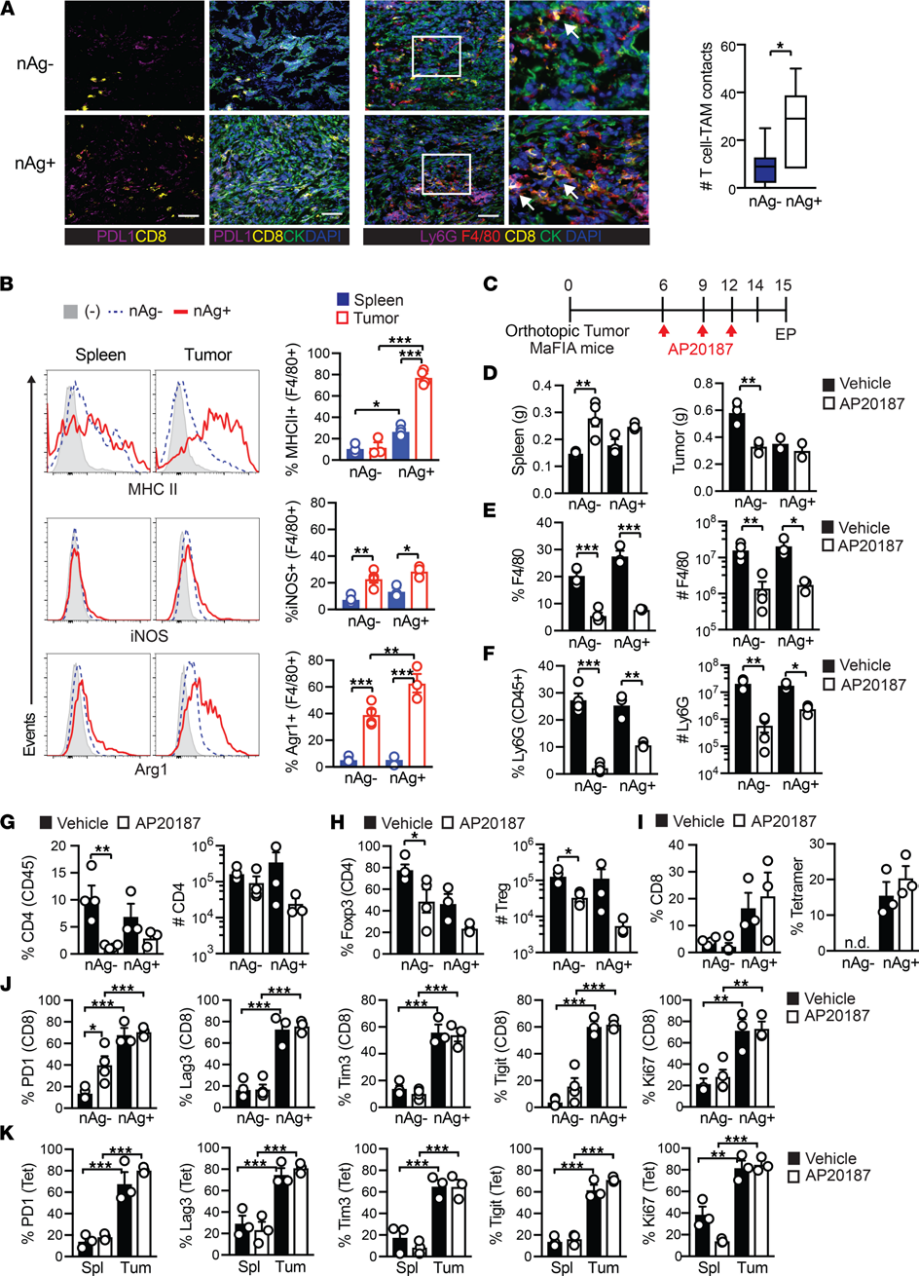
Protumor roles for TAMs and granulocytic myeloid-derived suppressor cells are obviated in neoantigen^+^ PDA. (**A**) Immunofluorescence staining of CB^+^ (nAg^+^) or CB^–^ (nAg^–^) orthotopic tumors on day 21. Arrows show CD8^+^ T cell contact with a macrophage. Scale bar: 50 μm. Original magnification of insets, 2.25×. The number of CD8^+^ T cell and TAM (F4/80^+^) contacts per field of view. The box plots depict the minimum and maximum values (whiskers), the upper and lower quartiles, and the median. The length of the box represents the interquartile range. (**B**) Proportion of splenic and intratumoral F4/80^+^ macrophages that express MHC II, iNOS, or Arg1 on day 14 posttumor. (**C**) Schematic for TAM depletion in MaFIA tumor-bearing mice. (**D**) Spleen and tumor weights on day 15. (**E**) Splenic F4/80^+^ cell proportion (of CD45^+^ cells) and number (normalized to tissue gram) from control or AP20187-treated MaFIA mice. (**F**) Intratumoral Ly6G^+^ frequency gated on CD45^+^ cells and cell number per gram tumor tissue. (**G**) Intratumoral CD4^+^ T cell frequency gated on CD45^+^ cells and number per gram tumor. (**H**) Intratumoral Treg frequency (of CD4^+^ cells) and number per gram tissue. (**I**) Intratumoral CD8^+^ T cell frequency gated on CD45^+^ (left) and CB_101-109_-specific T cell frequency gated on CD8^+^ T cells (right). n.d., not detected. (**J**) PD-1, Lag3, Tim3, Tigit, or Ki67 gated on total intratumoral CD8^+^ T cells. (**K**) Expression of PD-1, Lag3, Tim3, Tigit, or Ki67 gated on CB_101-109_:H-2D^b^ tetramer^+^ CD8^+^ T cells isolated from spleen (Spl) or tumor (Tum) from nAg^+^ tumor-bearing mice on day 15. In graphs, each dot is an independent mouse, data are mean ± SEM, and *n* = 3–5 mice per group and representative of 2 independent experiments. One-way ANOVA with a Tukey’s posttest (**B** and **G**–**K**) or Student’s *t* test to compare 2-group data (**A**) and 2-group data within each similar tumor-bearing cohort (**D**–**F**). *P < 0.05, **P < 0.005, and ***P < 0.0005.

We previously developed *KPC* orthotopic PDA mouse models that differ in CB model neoantigen (nAg) expression, resulting in T cell poor (nAg^–^) or T cell rich (nAg^+^) tumors (36) and differential responses to immunotherapy (36, 37). F4/80^+^ TAMs were abundant in both nAg^+^ and nAg^–^ tumors and colocalized with intratumoral CD8^+^ T cells (Figure 1A), consistent with our prior analysis of human PDA (7). TAMs isolated from nAg^+^ tumors expressed higher MHC II and arginase 1 (Arg1) compared with nAg^–^ tumors, whereas inducible nitric oxide synthase (iNOS) was similarly increased in TAMs from both nAg^+^ and nAg^–^ tumors compared with splenic macrophages (Figure 1B). Tumor cell–intrinsic differences in Myc, Notch, and chemokine pathways may affect T cell infiltration into independently derived *KPC* tumors (32). Although the nAg^+^ *KPC2a* tumor line is derived from the parental nAg^–^ *KPC2* tumor line (36), *Myc* and *Cdkn2a* appeared elevated in nAg^–^ versus nAg^+^ tumor cells, while various chemokines and *Notch1–4* were similarly expressed (Supplemental Figure 1A; supplemental material available online with this article; https://doi.org/10.1172/jci.insight.151593DS1). Although our results support that tumor antigenicity alters TAM phenotype, we cannot entirely exclude a role for tumor cell–intrinsic factors.

Due to the difference in TAM phenotype, we next tested if TAMs exhibit a differential role in nAg^–^ versus nAg^+^ tumors. Although we previously used anti–CSF1 receptor (αCSF1R) to deplete TAMs, this required repetitive and high dosing (46). We therefore employed macrophage Fas-induced apoptosis (MaFIA) mice (47), in which *Csf1r* drives expression of a mutant human FK506 binding protein 1A permitting the inducible apoptosis of Csf1r^+^ TAMs following treatment with AP20187. We orthotopically implanted nAg^+^ or nAg^–^ *KPC* tumor cells into the pancreas of MaFIA mice and treated cohorts with vehicle or AP20187 (Figure 1C). AP20187 significantly decreased nAg^–^ tumor size yet did not affect nAg^+^ tumor size (Figure 1D). Spleen size was significantly increased in mice bearing nAg^–^ tumors following AP20187 (Figure 1D). AP20187 significantly decreased the proportion and number of splenic macrophages in both tumor models (Figure 1E). Flow cytometric analysis suggested that the increase in spleen size following AP20187 (Figure 1D) was due to monocyte expansion (Supplemental Figure 1B). Unexpectedly, AP20187 also depleted Ly6G^+^ granulocytes, which contain granulocytic MDSCs (Gr-MDSCs) (14) (Figure 1F). Consistent with our results, a Csf1r reporter indicated Ly6G^+^ cells expressed low Csf1r (48). AP20187 significantly decreased the proportion of total CD4^+^ T cells (Figure 1G) and CD4^+^Foxp3^+^ Tregs in nAg^–^ tumors (Figure 1H). However, Csf1r was not detected on CD4^+^ T cells (Supplemental Figure 1C), suggesting that the decrease in Tregs following AP20187 was indirect and trended to decrease the intratumoral CD4^+^/CD8^+^ T cell ratio (Supplemental Figure 1D). However, neither AP20187 treatment nor the resulting Treg depletion impacted overall CD8^+^ T cell frequency in nAg^–^ or nAg^+^ tumors, as well as tumor-specific T cell frequency in nAg^+^ tumors (Figure 1I). In contrast to nAg^–^ tumors, most CD8^+^ T cells in nAg^+^ tumors expressed PD-1, Lag3, Tim3, Tigit, and Ki67 (Figure 1J), consistent with sustained antigen recognition and exhausted T cell (T_EX_) differentiation (36, 37). While AP20187 treatment increased PD-1^+^CD8^+^ T cells in nAg^–^ tumors (Figure 1J), additional coinhibitory receptors Lag3, Tim3, and Tigit were not increased (Figure 1J). This treatment also did not further increase inhibitory receptors on intratumoral tetramer^+^ T cells (Figure 1K). Thus, putative immunosuppressive myeloid cells do not appear to contribute to T_EX_ differentiation of tumor-specific T cells, at least within the confines of this approach and time point.

### Neoantigen promotes cDC1 accumulation in PDA.

**Figure 2:**
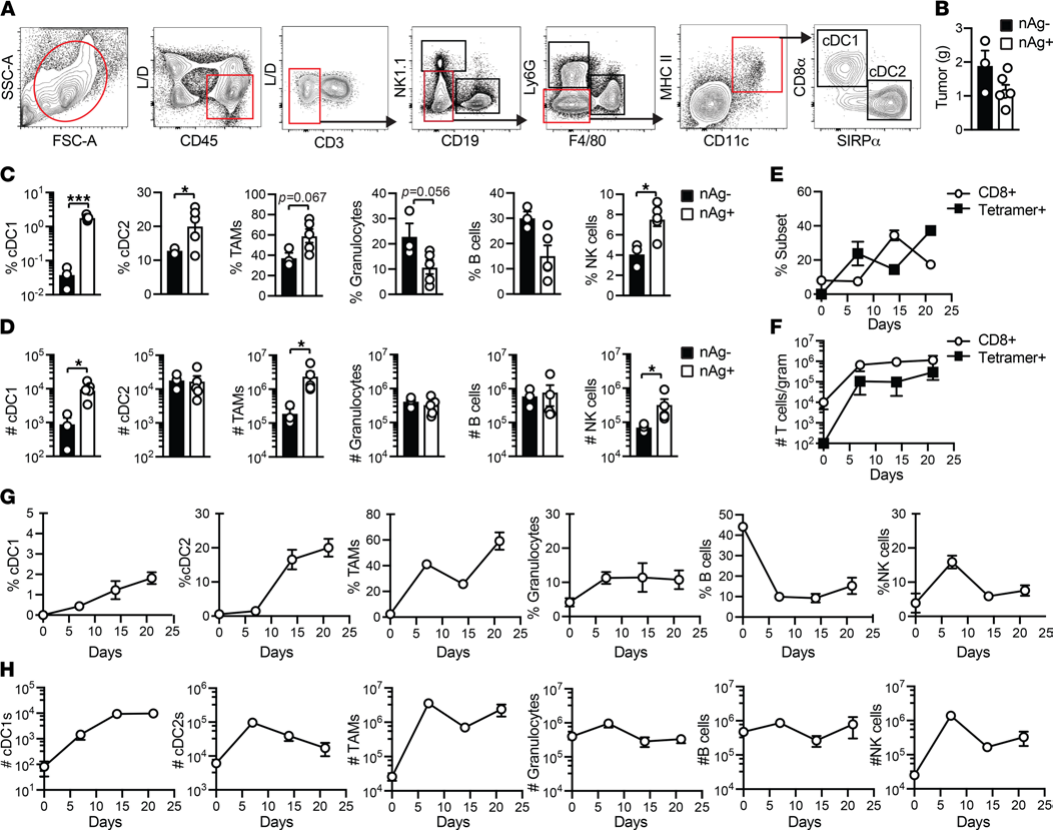
Neoantigen promotes cDC1 accumulation in PDA. (**A**) Splenic and intratumoral mononuclear cells were gated on live, CD45^+^CD3^–^ cells, followed by excluding CD19^+^ B cells and NK1.1^+^ NK cells. Ly6G^+^ granulocytes and F4/80^+^ macrophages were next excluded. CD3^–^CD19^–^NK1.1^–^Ly6G^–^F4/80^–^ cells were next gated on CD11c^+^MHC II^+^ DCs, which were further divided into CD8α^+^ cDC1s and SIRPα^+^ cDC2s. This gating strategy was also used to identify macrophages and granulocytes in Figure 1. (**B**) nAg^+^ and nAg^–^ orthotopic tumor weights on day 21. (**C**) Frequency of immune cell subsets among CD45^+^ cells in nAg^+^ and nAg^–^ tumors on day 21. Unpaired 2-tailed Student’s *t* test. (**D**) Number of immune cell subsets in nAg^+^ and nAg^–^ tumors normalized to tumor weight. Unpaired 2-tailed Student’s *t* test. Frequency (**E**) and number (**F**) of CD8^+^ T cells gated on CD45^+^ cells and tetramer^+^ (CB_101-109_:H-2D^b^) T cells gated on CD8^+^ T cells in nAg^+^ tumors. (**G**) Kinetics of immune cell frequency gated on CD45^+^ cells in nAg^+^ tumors. (**H**) Kinetics of cell number normalized to gram tumor. (**B**–**D**) Each dot is an independent mouse. All graphed data are mean ± SEM. *n* = 3–5 mice per group and representative of 2–3 independent experiments. *P < 0.05, and ***P < 0.0005.

PDA can interfere with cDC1 differentiation and accumulation (9–11). Therefore, we next set out using the gating strategy in Figure 2A to determine if antigenicity impacts cDC1s and/or cDC2s (49, 50). cDC1s isolated from spleen and orthotopic tumors coexpressed CD8α and Xcr1 (Supplemental Figure 2). We selected day 21 after tumor implantation (posttumor) because tumor size was comparable (Figure 2B). We detected a significant increase in cDC1, cDC2, and NK cell frequency and trends for increased TAM and decreased Gr-MDSC and B cell frequency in nAg^+^ versus nAg^–^ tumors (Figure 2C). nAg^+^ tumors contained more cDC1s, TAMs, and NK cells normalized to gram of tumor tissue (Figure 2D).

We next performed a kinetic analysis of immune cells in nAg^+^ tumors. Intratumoral CD8^+^ T cells and tetramer^+^ T cell frequencies fluctuated (Figure 2E). However, overall CD8^+^ T cell and tumor-specific T cell number remained constant between 1 and 3 weeks posttumor (Figure 2F). cDC1 proportion increased over time, resulting in a significant increase in cDC1 number on days 14 (*P* = 0.012) and 21 (*P* = 0.006) in tumors compared with healthy pancreas prior to tumor implantation (day 0, Figure 2, G and H). cDC2 and TAM cell number significantly increased on day 7 after (*P* = 0.0001 and *P* = 0.0063, respectively, Figure 2H). In contrast, neither Gr-MDSC nor B cell frequency or number increased during nAg^+^ tumor growth (Figure 2, G and H). NK cells increased by almost 2 logs on day 7 and rapidly decreased at later time points (Figure 2, G and H). The data suggest that tumor antigenicity, and therefore probably tumor-specific T cells, may overcome PDA-mediated subversion of cDC1s.

### Effective immunotherapy regimens expand splenic cDC1s.

**Figure 3:**
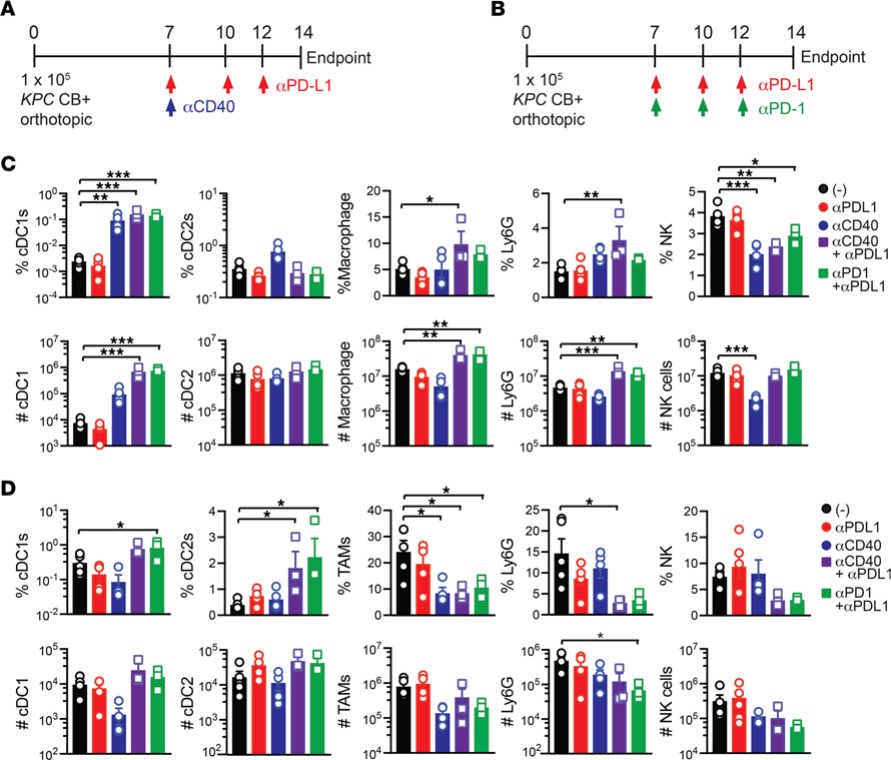
Effective immunotherapy regimens expand splenic cDC1s. Schematic for testing agonistic αCD40 + αPD-L1 (**A**) or αPD-L1 + αPD-1 (**B**) on immune cell composition in nAg^+^ orthotopic tumors. (**C**) Frequency (top row, gated on live CD45^+^ cells) and number (bottom row, normalized to tissue gram) of immune cells in spleen from control or immunotherapy-treated mice. (**D**) Frequency (top row, gated on live CD45^+^ cells) and number (bottom row, normalized to tissue gram) of immune cells in tumor from control or immunotherapy-treated mice. Each dot is an independent animal. Data are mean ± SEM and *n* = 4–6 mice per group and 2 independent experiments. *P < 0.05, **P < 0.005, ***P < 0.0005. One-way ANOVA with a Tukey’s posttest.

We previously identified that combinations of αPD-1+αPD-L1 (36) or agonistic αCD40+αPD-L1 (37) exhibited greater antitumor activity as compared with monotherapies in the nAg^+^ model. αPD-L1 expanded tumor-specific T cells in the spleen (36) whereas αCD40 reduced intratumoral regulatory cytokines, reduced T_EX_, and promoted Klrg1^+^ cytotoxic intratumoral T cells (37). PD-L1 was expressed by both splenic and intratumoral granulocytes, cDC1s, and cDC2s and elevated on TAMs as compared with splenic macrophages (Supplemental Figure 3, A and B). CD40 was expressed by splenic and intratumoral B cells, macrophages, cDC1s, and cDC2s and was increased on TAMs as compared with macrophages isolated from healthy pancreas (day 0, Supplemental Figure 3, A and B). We next tested if immunotherapy regimens impact the myeloid cell infiltrate (Figure 3, A and B) (36, 37). We selected 2 weeks posttumor because tumor size is not significantly different among the cohorts (37). Combination immunotherapies markedly increased splenic cDC1 frequency, resulting in a 2-log increase in splenic cDC1 number (Figure 3C and Supplemental Figure 3C). Combination regimens also increased splenic macrophage and granulocyte number (Figure 3C). The regimens trended toward an increase in cDC1 frequency and number in tumors, while concomitantly reducing TAMs and granulocytes (Figure 3D). Agonistic αCD40 reduced NK cell frequency and number in spleen (Figure 3C). Thus, while immunotherapy induces multiple cellular changes, both combinations markedly expand splenic cDC1s.

### Batf3 is required for tumor-specific T cell priming to a pancreas tumor antigen.

**Figure 4:**
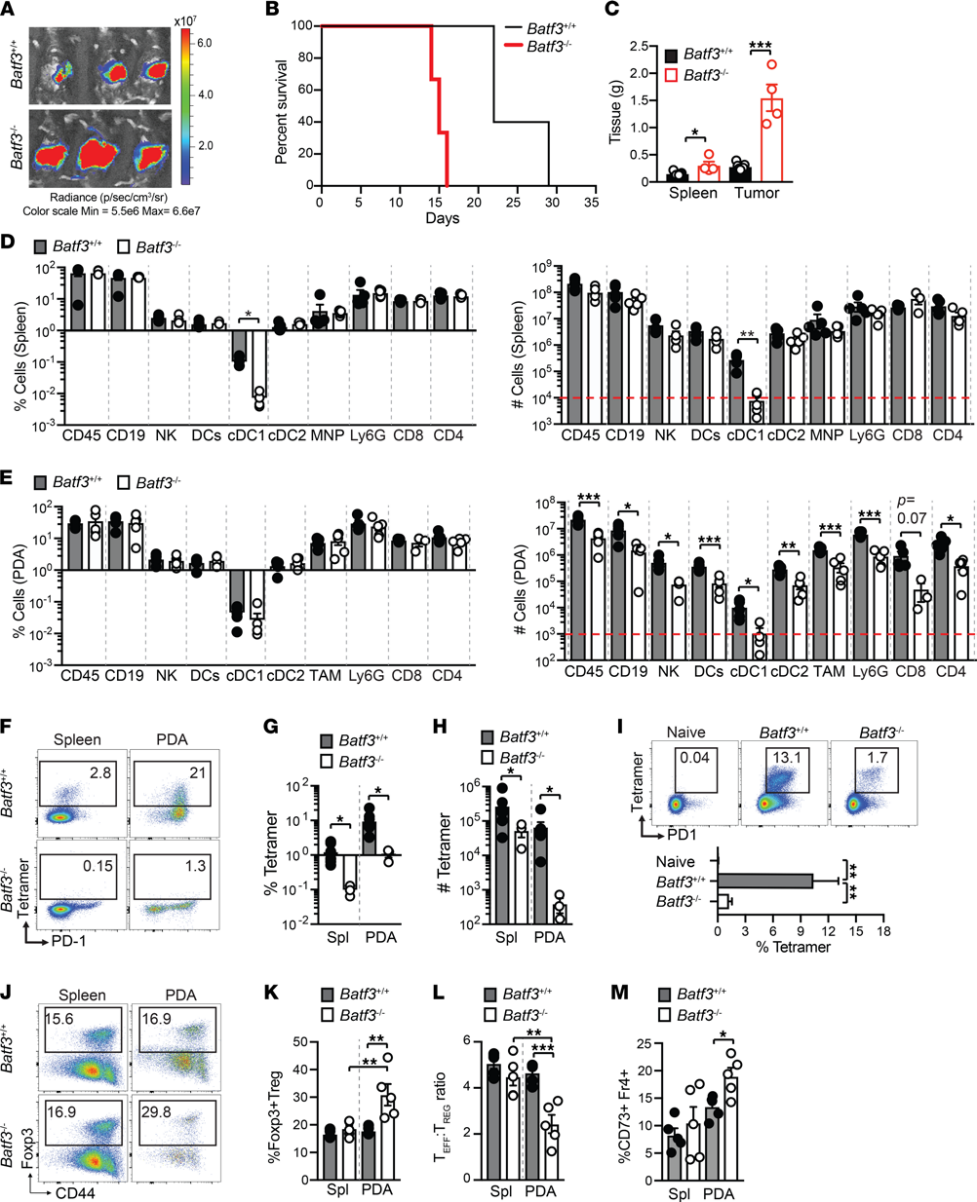
*Batf3* is required for tumor-specific T cell priming to a pancreas tumor antigen. (**A**) Tumor radiance at 7 days posttumor in *Batf3*^+/+^ and *Batf3*^–/–^ mice. (**B**) Survival of *Batf3*^+/+^ or *Batf3*^–/–^ mice bearing nAg^+^ tumors. Log-rank (Mantel-Cox) test, *n* = 4–6 mice per group. (**C**) Spleen and orthotopic tumor weights from *Batf3*^+/+^ and *Batf3*^–/–^ mice at day 14 posttumor. Student’s *t* tests. (**D**) Frequency of immune cells in spleen gated on CD45^+^ cells (left) and cell number normalized to spleen gram (right) at day 14. Student’s *t* test. Dotted red line indicates limit of detection. (**E**) Frequency of intratumoral immune cells gated on CD45^+^ cells (left) and cell number normalized to tumor gram (right) at day 14. Student’s *t* test. Dotted red line indicates limit of detection. (**F**) Representative CB_101-109_:H-2D^b^ tetramer staining gated on CD8^+^ T cells at day 14. (**G**) Frequency of CB_101-109_:H-2D^b^-specific T cells at day 14. Student’s *t* test. (**H**) Number of CB_101-109_:H-2D^b^-specific T cells isolated from spleen (Spl) or tumors (PDA) and normalized to tissue gram on day 14. Student’s *t* test. (**I**) Representative plots of CB_101-109_:H-2D^b^-specific T cell tetramer and PD-1 gated on CD8^+^ T cells of spleens of naive mice or mice vaccinated with CB_101-109_ peptide, CD40 agonist, and Poly:(IC) on day 7. Data are graphed below. One-way ANOVA with Tukey’s posttest. (**J**) Foxp3 and CD44 staining of live CD45^+^CD4^+^ T cells isolated from spleens and tumors at day 14. (**K**) Frequency of Foxp3^+^CD4^+^ T cells gated on total CD4^+^ T cells in spleen (Spl) and tumor (PDA). Student’s *t* test. (**L**) Effector CD8^+^ T cell/Treg ratio in tumors from *Batf3*^+/+^ and *Batf3*^–/–^ mice. (**M**) Frequency of CD4^+^Foxp3^–^ T cells that coexpress Fr4 and CD73. (**C**–**E**, **G**, **H**, and **K**–**M**) Each dot is an independent mouse. Data are mean ± SEM. *n* = 3–6 mice per group and 2–3 independent experiments. *P < 0.05, **P < 0.005, and ***P < 0.0005.

To test the role of cDC1s on tumor-specific T cell priming in PDA, we orthotopically implanted nAg^+^ tumor cells into the pancreas of *Batf3*^+/+^ or *Batf3*^–/–^ mice, which lack cDC1s (15–19). We observed fulminant tumor growth in *Batf3*^–/–^ mice (Figure 4A) and decreased survival (Figure 4B). Both tumor and spleen size were significantly larger in *Batf3*^–/–^ than *Batf3*^+/+^ mice at day 14 posttumor (Figure 4C). As anticipated, splenic cDC1s were below the limit of detection in *Batf3^–/–^* mice (Supplemental Figure 4A and Figure 4D), consistent with prior research (18, 19). Intratumoral immune cell frequency was similar in *Batf3^–/–^* and *Batf3*^+/+^ mice (Figure 4E). However, immune cell number normalized to tumor gram was dramatically decreased in *Batf3*^–/–^ mice (Figure 4E), which was probably due to increased tumor size (Figure 4C). Tumor-specific T cells were undetectable in *Batf3*^–/–^ tumor-bearing mice (Figure 4, F–H). IFN-γ production following ex vivo peptide restimulation was also undetectable in *Batf3*^–/–^ mice (Supplemental Figure 4B). To investigate if a CD8^+^ T cell response could be generated in *Batf3^–/–^* mice if crosspresentation was overcome, we immunized *Batf3^–/–^* or *Batf3*^+/+^ mice with CB_101-109_ peptide, agonistic αCD40, and Poly(I:C) (36). Antigen-specific T cells expanded in *Batf3*^–/–^ mice, yet this was significantly less than *Batf3*^+/+^ mice (Figure 4I). *Batf3*^–/–^ mice also contained a higher proportion of intratumoral Tregs than *Batf3*^+/+^ mice (Figure 4, J and K), resulting in a significantly lower effector T cell (Teff) to Treg ratio (Figure 4L), which may reflect Batf3 repression of Foxp3 in CD4^+^ T cells (26, 29). Tumors from *Batf3*^–/–^ mice were also enriched for CD4^+^Foxp3^–^CD73^+^Fr4^+^ T cells (Figure 4M and Supplemental Figure 4C), an anergic phenotype (51).

### Xcr1 signaling mediates splenic and intratumoral cDC1 accumulation.

**Figure 5:**
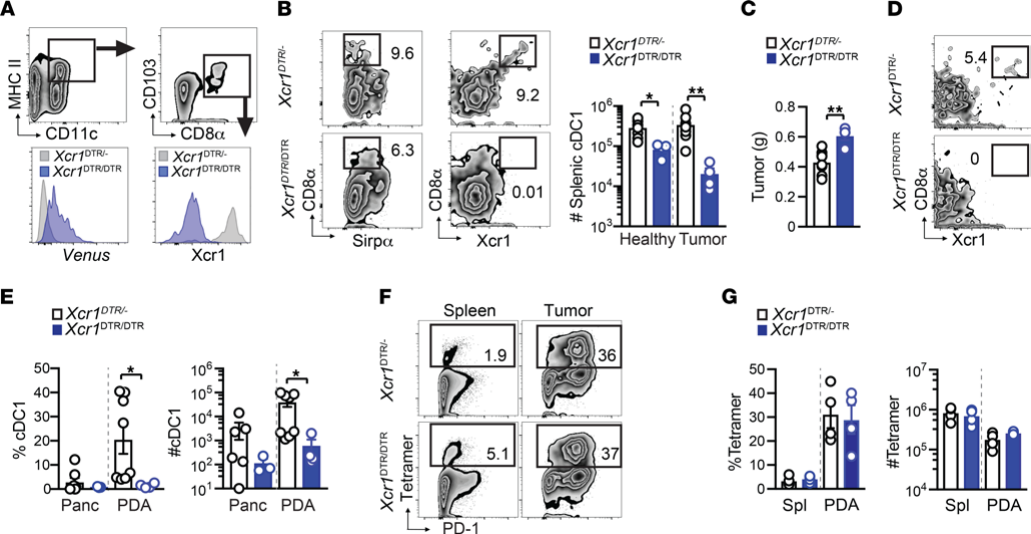
Xcr1 signaling mediates splenic and intratumoral cDC1 accumulation. (**A**) Gating strategy and Xcr1 expression by splenic cDC1s isolated from unmanipulated *Xcr1*^DTR/–^ or *Xcr1*^DTR/DTR^ mice. Histogram overlays represent the Venus reporter (left) and Xcl1 antibody staining (right). (**B**) Representative flow cytometric plots of spleen cells gated on CD11c^+^MHC II^+^ DCs on day 14 after orthotopic CB^+^ tumor implantation. Quantification of splenic cDC1 number in normal or day 14 tumor-bearing mice. (**C**) Orthotopic tumor weight in grams at day 14. Unpaired 2-tailed Student’s *t* test. (**D**) Representative plots of intratumoral cDC1s from *Xcr1*^DTR/–^ or *Xcr1*^DTR/DTR^ mice at day 14. Gated on CD11c^+^MHC II^+^ DCs. (**E**) Proportion and number of cDC1s isolated from healthy pancreas (Panc) or from orthotopic tumors at day 14. Data are mean ± SEM, Student’s *t* test. (**F**) Frequency of splenic and intratumoral CD8^+^ T cells that bind CB_101-109_:H-2D^b^ tetramer at day 14. Plots are gated on live, CD45^+^CD8^+^ T cells. (**G**) Frequency (left) and number (right) of CB_101-109_:H-2D^b^-specific CD8^+^ T cells in spleen (Spl) and tumors at day 14 after tumor implantation. Student’s *t* test. (**B**–**E** and **G**) Each dot is an independent mouse. Data are mean ± SEM and pooled from 2 independent experiments. *n* = 4–7 mice per group. *P < 0.05, and **P < 0.005.

We next sought to identify the mechanism(s) driving cDC1 accumulation during tumor growth. Since both lymph node resident and nonresident cDC1s express Xcr1 (52), we employed *Xcr1*^DTRvenus^ homozygous mice (*Xcr1*^DTR/DTR^), which have the coding region for *Xcr1* replaced with a diphtheria toxin (DT) receptor linked to a Venus fluorescent reporter (30). Since cDC1s from *Xcr1*^DTR/DTR^ mice lack Xcr1 (Figure 5A and ref. 30), we first tested if Xcr1 loss impacted cDC1 abundance without DT administration. Splenic cDC1s were significantly reduced about 2-fold in healthy, and about 10-fold in orthotopic tumor-bearing, *Xcr1*^DTR/DTR^ mice compared with *Xcr1*^DTR/–^ mice (Figure 5B). Xcr1 deletion increased tumor size (Figure 5C) and caused a significant reduction in intratumoral cDC1 frequency and number (Figure 5, D and E). Regardless, tumor-specific T cells expanded in the absence of Xcr1 (Figure 5, F and G), suggesting that cDC1s that lack Xcr1 still elicit tumor-specific T cells.

**Figure 6:**
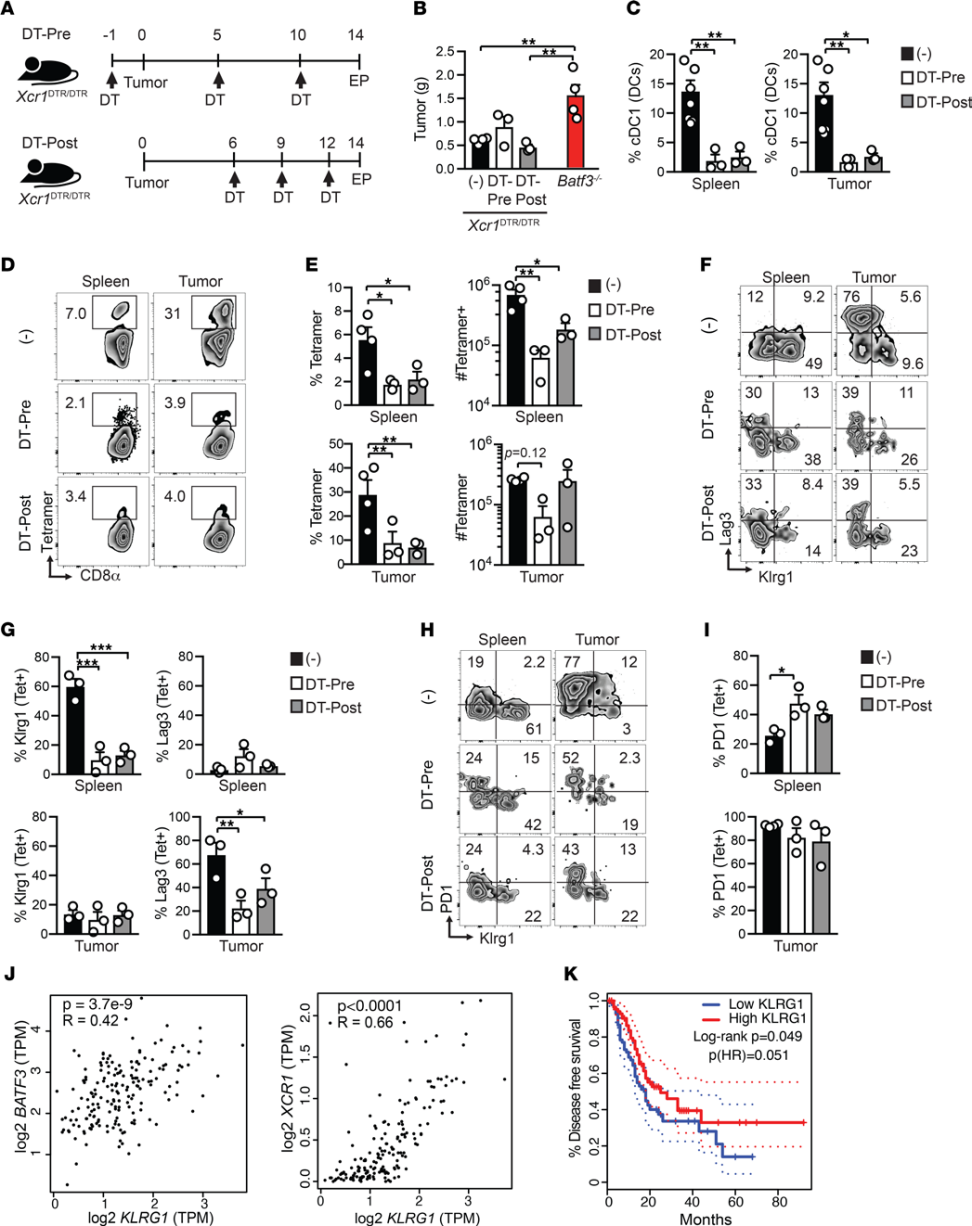
cDC1s promote endogenous splenic Klrg1^+^ and intratumoral Lag3^+^ tumor-specific T cells. (**A**) Schematic of diphtheria toxin (DT) administration in orthotopic tumor-bearing *Xcr1*^DTR/DTR^ mice to deplete cDC1s prior to (DT-pre) or following (DT-post) tumor implantation. EP, endpoint. (**B**) Tumor weights isolated from untreated *Xcr1*^DTR/DTR^ mice, *Xcr1*^DTR/DTR^ mice treated with DT either prior to (Pre) or after tumor implantation, and *Batf3*^–/–^ mice on day 14. One-way ANOVA with a Tukey’s posttest. (**C**) cDC1 frequency among CD11c^+^MHC II^+^ DCs in control or DT-treated *Xcr1*^DTR/DTR^ mice. One-way ANOVA with a Tukey’s posttest. (**D**) Representative CB_101-109_:H-2D^b^ tetramer staining gated on CD8^+^ T cells from untreated or DT-treated *Xcr1*^DTR/DTR^ mice. (**E**) Quantification of **D**. One-way ANOVA with a Tukey’s posttest. (**F**) Representative Lag3 and Klrg1 staining by CD8^+^tetramer^+^ T cells. (**G**) Quantification of **F**. One-way ANOVA with a Tukey’s posttest. (**H**) Representative PD-1 and Lag3 staining by CD8^+^tetramer^+^ T cells. (**I**) Quantification of **H**. One-way ANOVA with a Tukey’s posttest. (**J**) Correlation between *KLRG1* and cDC1 genes in 176 human PDAs from The Cancer Genome Atlas (TCGA) data set was determined using GEPIA (http://gepia.cancer-pku.cn/). (**K**) Patient tumors with high *KLRG1* transcripts per million (TPM) have significantly improved disease-free survival. 176 PDAs from the TCGA data set were divided into *KLRG1*-high (*n* = 88) or *KLRG1*-low (*n* = 88) expressers and survival outcomes determined using GEPIA. Graphed data are mean ± SEM. *n* = 4 mice per group. Representative of 2 independent experiments. *P < 0.05, **P < 0.005, and ***P < 0.0005.

Our prior studies support that Klrg1 is a surrogate marker for IFN-γ^+^Granzyme B^+^ functional Teffs, contrasting with dysfunctional Klrg1^–^Lag3^+^ T_EX_ in nAg^+^ PDA (36, 37). Most fully functional splenic tumor-specific T cells express Klrg1 whereas intratumoral tumor-specific T cells rapidly lose Klrg1 and upregulate Lag3 (36, 37). T_EX_ exhibit a progressive failure to produce IFN-γ and Granzyme B after in vitro peptide stimulation (36, 37). Combination immunotherapies (Figure 3) increase intratumoral functional Klrg1^+^Lag3^–^ T cells while decreasing Klrg1^–^Lag3^+^ T_EX_ (36, 37). Similar to antigen-specific T cell responses (36, 37), intratumoral Klrg1^+^Lag3^–^CD8^+^ T cells were highly functional as measured by IFN-γ and Granzyme B, whereas Klrg1^–^Lag3^+^CD8^+^ T cells were less functional following PMA/ionomycin stimulation (Supplemental Figure 5A). To determine if cDC1s influenced these T cell subsets, we used the conditional cDC1 depletion approach because tumor-specific T cells are not detectable in *Batf3*^–/–^ mice (Figure 4). Conditional cDC1 depletion significantly decreased splenic Klrg1^+^ and intratumoral Lag3^+^ tumor-specific T cell frequency (Figure 6, F and G). cDC1 depletion prior to tumor implantation significantly increased splenic but not intratumoral PD-1^+^ tumor-specific T cell frequency (Figure 6, H and I). In human PDA, cDC1 genes correlated with *KLRG1* (Figure 6J) as well as *CD8A*, *CD8B*, and *GZMA* (Supplemental Figure 5B). *KLRG1* also significantly correlated with prolonged disease-free survival in patients with PDA (Figure 6K). While the role for cDC1s influencing prototypical exhaustion markers is less clear, cDC1s promote endogenous Klrg1^+^ tumor-specific T cell expansion and thus a reservoir of functional tumor-specific T cells.

### Host cDC1s reactivate transferred effector and memory T cells to promote splenic Klrg1^+^ T cells and tumor control.

**Figure 7:**
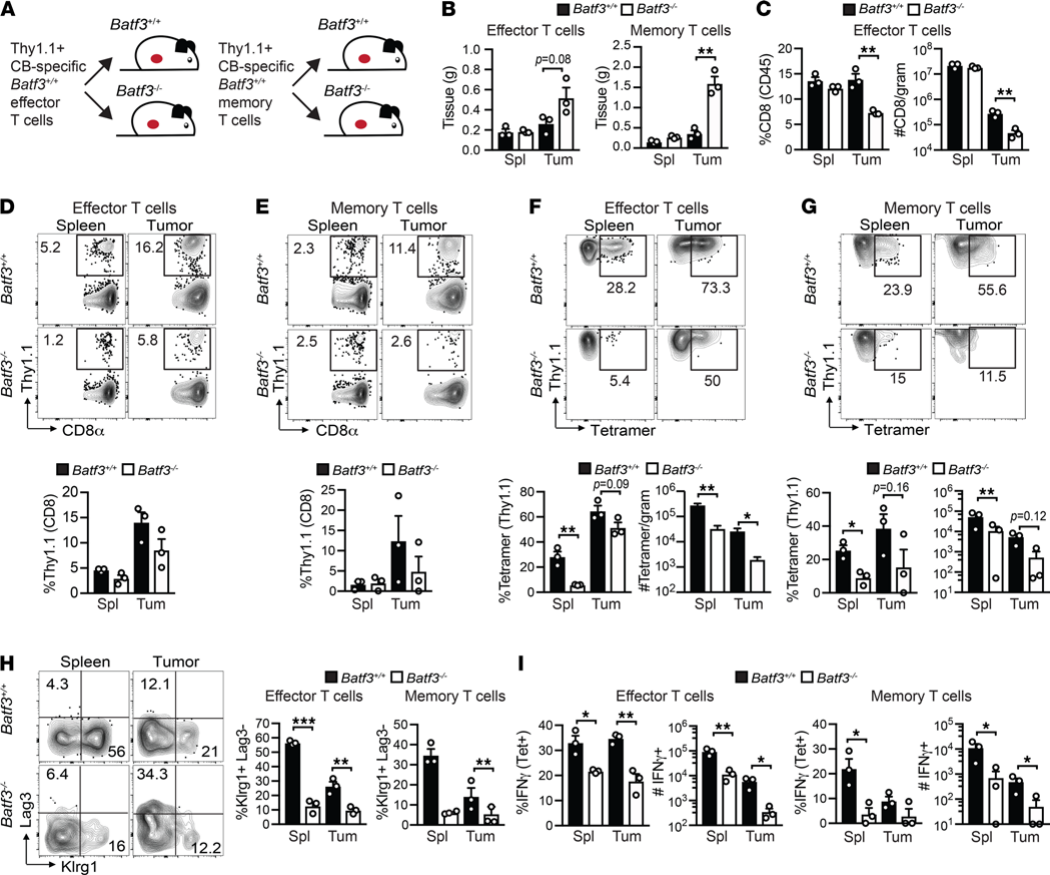
Host cDC1s reactivate transferred effector and memory T cells to promote splenic Klrg1^+^ T cells and tumor control. (**A**) Simplified schematic of effector or memory CB_101-109_ -specific CD8^+^ T cell transfers into *Batf3^+/+^* or *Batf3^–/–^* tumor-bearing mice. (**B**) Spleen (Spl) and tumor (Tum) weights in grams (g) on day 14 after memory T cell transfer and day 7 after effector T cell transfer. (**C**) Frequency and number of CD8^+^ T cells in recipients of effector T cells. For memory T cells, see Supplemental Figure 6A. Frequency of Thy1.1^+^ effector (**D**) and memory (**E**) T cells in recipients gated on live CD8^+^ T cells. Data are quantified below plots. Frequency of CD8^+^Thy1.1^+^tetramer^+^ effector (**F**) and memory (**G**) T cells in recipients. Plots are gated on live CD8^+^Thy1.1^+^ T cells. Data are quantified below plots. (**H**) Representative plots of Klrg1 and Lag3 by CD8^+^Thy1.1^+^tetramer^+^ T cells and quantified data. (**I**) Cytokine secretion by CD8^+^Thy1.1^+^ in response to stimulation with CB_101-109_ peptide in vitro was determined by intracellular cytokine staining. All graphed data are mean ± SEM. *n* = 3 mice per T cell recipient group. Representative of 2 independent experiments. *P < 0.05, **P < 0.005, and ***P < 0.0005. Student’s *t* test was used for analysis of 2-group data.

We next tested the requirement for cDC1s at the effector and memory phases of T cell differentiation. To overcome any CD8^+^ T cell–intrinsic role for *Batf3* as has been reported for memory T cells (27, 53), as well as incomplete cDC1 depletion in *Xcr1*^DTR/DTR^ mice (Figure 6C), we pursued an alternative approach. We generated CB-specific effector and memory T cells by immunizing Thy1.1^+^*Batf3^+/+^* mice with CB_101-109_, αCD40, and Poly(I:C). Next, splenocytes from Thy1.1^+^ Teffs were isolated 7 days after vaccination and transferred into day 3 tumor-bearing *Batf3^+/+^* or *Batf3^–/–^* mice. Memory Thy1.1^+^ T cells were isolated 41 days after vaccination and transferred into *Batf3^+/+^* or *Batf3^–/–^* mice on the same day mice received orthotopic CB^+^ tumors (Figure 7A). We selected early time points for the T cell transfer to minimize differences in tumor size that occur in *Batf3^+/+^* and *Batf3^–/–^* mice (Figure 4C). Transferred Teffs and memory T cells exhibited greater tumor control in *Batf3^+/+^* mice compared with *Batf3^–/–^* mice (Figure 7B). Both frequency and number of total CD8^+^ T cells were decreased in tumors but not spleens in *Batf3^–/–^* recipients of Teffs (Figure 7C) but not memory T cells (Supplemental Figure 6A). Thy1.1^+^ Teff or memory T cell frequency appeared decreased in tumors in *Batf3^–/–^* compared with *Batf3^+/+^* mice (Figure 7, D and E). Notably, there was a significant decrease in both frequency and number of donor CD8^+^tetramer^+^ Teff and memory T cells in both the spleen and tumor of *Batf3^–/–^* recipients (Figure 7, F and G).

To analyze the role of host cDC1s in T cell differentiation, we evaluated the markers Klrg1 and Lag3 as these distinguish potent effector T cells from T_EX_ (36, 37) (Supplemental Figure 5). We observed a dramatic decrease in donor Klrg1^+^tetramer^+^ T cells in *Batf3^–/–^* compared with *Batf3^+/+^* recipients (Figure 7H). Both donor Teffs and memory T cells were deficient in IFN-γ in *Batf3^–/–^* recipients (Figure 7I and Supplemental Figure 6B). Thus, host cDC1s are critical for reactivation and expansion of both Teffs and memory T cells. Notably, cDC1s are also key drivers of the differentiation of Klrg1^+^IFN-γ^+^ T cells, which our prior studies show are particularly critical for tumor control (36, 37).

### CD40 agonist primes tumor-specific CD8^+^ T cells via monocytes/macrophages in Batf3^–/–^ mice.

**Figure 8:**
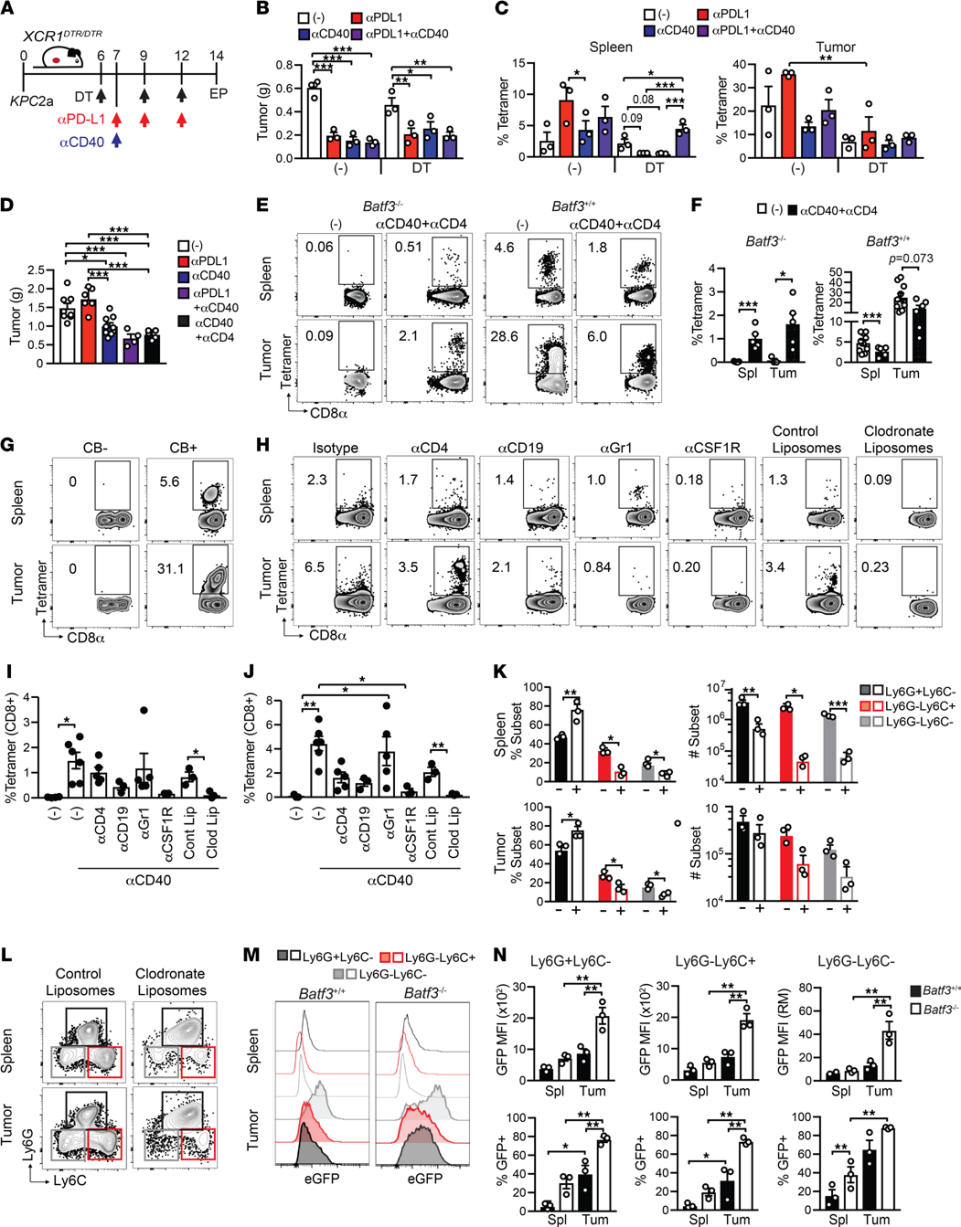
CD40 agonist exhibits partial antitumor activity and primes tumor-specific CD8^+^ T cells via monocytes/macrophages in *Batf3^–/–^* mice. (**A**) Experimental schematic. EP, endpoint. (**B**) Orthotopic tumor weights on day 14. (**C**) Proportion of splenic (left) and intratumoral (right) CD8^+^ T cells that bind CB_101-109_:H-2D^b^ tetramer. There were no significant differences in tumor size between control and cDC1-depleted cohorts. (**D**) Tumor weights in treated or control *Batf3*^–/–^ mice on day 14. (**E**) Representative of tetramer^+^ T cell frequency gated on CD8 T cells. (**F**) Quantification of **E**. (**G**) Frequency of tetramer^+^ T cells from orthotopic CB^–^ or CB^+^ tumors on day 14 posttumor. Representative of *n* = 10 mice. (**H**) Frequency of CD8^+^tetramer^+^ T cells from *Batf3*^–/–^ mice treated with CD40 agonist and various cell depletions. CD8^+^tetramer^+^ T cell frequency in spleen (**I**) and tumor (**J**). (**K**) Frequency (left) and number (right) of the indicated CD11b^+^ myeloid subsets in *Batf3*^–/–^ mice that received control liposomes (-) or clodronate liposomes (+) and were treated with CD40 agonist in spleen (top) and tumor (bottom). (**L**) Myeloid subsets are gated on live, CD45^+^CD11b^+^ cells. (**M**) Representative histograms of EGFP among myeloid subsets from *Batf3*^+/+^ and *Batf3*^–/–^ mice on day 7 after agonistic αCD40. (**N**) Graphed data from **M** in spleen (Spl) and tumor (Tum). All graphed data are mean ± SEM and each dot is an independent animal. Representative of at least 2 independent experiments. *P < 0.05, **P < 0.005, and ***P < 0.0005. One-way ANOVA with a Tukey’s posttest (**B**, **C**, **D**, **I**, **J**, and **N**); Student’s *t* test (**F** and **K**). *n* = 3–8 mice per group.

To test the role of cDC1s in response to treatment with αPD-L1 and/or agonistic αCD40, cDC1s were depleted 1 day prior to immunotherapy in tumor-bearing *Xcr1*^DTR/DTR^ mice (Figure 8A). The immunotherapies exhibited similar antitumor activity in cDC1-depleted hosts (Figure 8B). Like our prior studies, αPD-L1 expanded splenic tumor-specific T cells in control mice, which appeared cDC1 dependent (Figure 8C). In contrast to conditionally cDC1-depleted mice, αPD-L1 antitumor activity was completely abrogated in *Batf3^–/–^* mice (Figure 8D). Unexpectedly, CD40 agonist alone or in combination with αPD-L1 exhibited partial yet significant antitumor activity in *Batf3^–/–^* mice (Figure 8D). These data suggest that CD40 agonist operates, in part, via a cDC1-independent pathway. We reasoned that when CD8^+^ T cell responses are impaired, CD4^+^ T cells may provide an alternative antitumor mechanism. However, CD4^+^ T cell depletion failed to abrogate αCD40 activity (Figure 8D), and efficacy of T cell depletion was confirmed (Supplemental Figure 7A). Unexpectedly, agonistic αCD40 + αCD4 induced tumor-specific CD8^+^ T cell expansion in *Batf3*^–/–^ mice (Figure 8, E and F). As tetramer^+^ T cells are quite rare in cDC1-deficient hosts, specificity of the tetramer-binding T cells was validated by staining T cells from CB^–^ and CB^+^ tumors (Figure 8G). To identify the non-cDC1 antigen-presenting cell (APC) that primes tumor-specific CD8^+^ T cells, we depleted various immune cells in *Batf3^–/–^* mice prior to orthotopic tumor implantation and αCD40. Specificity and efficiency of immune cell–specific depletion of the various strategies at endpoint in spleen and tumors was quantified by flow cytometry (Supplemental Figure 7A). Notably, administration of clodronate liposomes, which deplete phagocytic cells, abrogated the expansion of CD8^+^tetramer^+^ T cells in both spleen and tumor (Figure 8, H and I). In contrast, neither B cell depletion nor αGr1 administration, which binds Ly6G on neutrophils and Ly6C to a lesser extent (54), abrogated T cell priming (Figure 8, H and I). Albeit not significant, macrophage depletion using αCSF1R trended to interfere with T cell priming in the spleen (Figure 8, H and I). CSF1R blockade also trended to decrease the frequency of intratumoral tetramer^+^ T cells (Figure 8, H and J, *P* = 0.077), suggesting TAMs may recruit and/or retain tumor-specific T cells.

In contrast to control liposomes, clodronate liposomes dramatically depleted Ly6C^hi^ classical and Ly6C^lo^CD11b^+^ nonclassical monocytes in both spleen and tumor (Figure 8, K and L). The loss of nonclassical monocytes could be secondary to the loss of classical monocytes because Ly6C^hi^ classical monocytes rapidly differentiate into Ly6C^lo^ nonclassical monocytes (55). Since the CB is linked to EGFP expression, we used EGFP to measure tumor antigen uptake in monocytes and granulocytes (Figure 8M). All 3 intratumoral myeloid subsets acquired tumor antigen in both *Batf3^+/+^* and *Batf3*^–/–^ hosts (Figure 8M). More cells acquired EGFP and expressed higher EGFP levels in *Batf3*^–/–^ than *Batf3^+/+^* hosts (Figure 8N), which could be due to greater tumor burden. Thus, our data suggest that αCD40 programs monocytes/macrophage to prime tumor-specific CD8^+^ T cells in cDC1-deficient hosts.

### αCD40-induced monocyte/macrophage priming generates atypical tumor-specific T cells that lack antitumor activity in Batf3^–/–^ mice.

**Figure 9:**
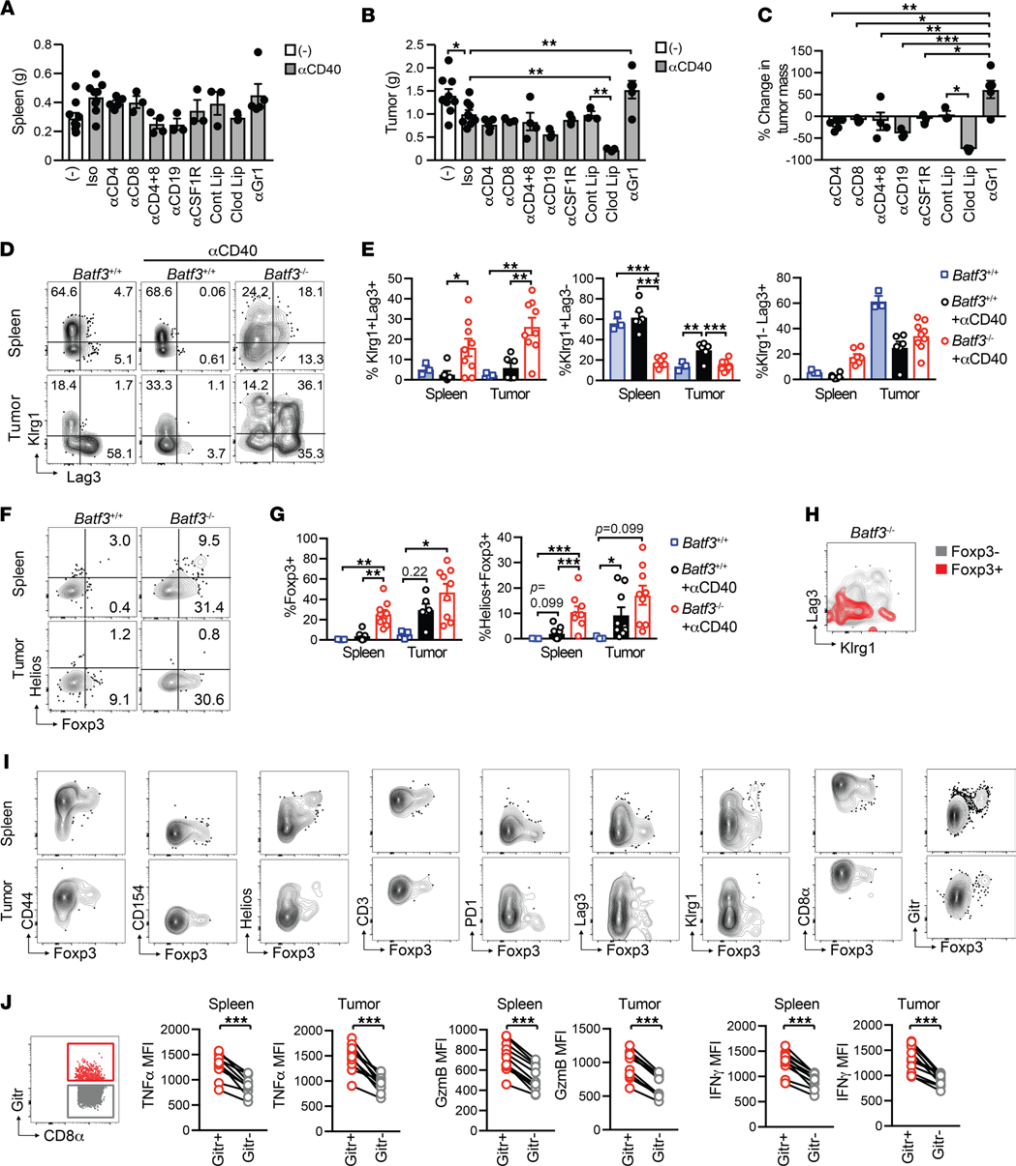
αCD40-induced monocyte/macrophage priming generates atypical tumor-specific T cells that lack antitumor activity in *Batf3*^–/–^ mice. (**A**) *Batf3*^–/–^ spleen weight after various cell depletions + CD40 agonist. (**B**) *Batf3*^–/–^ tumor weight after various cell depletions + CD40 agonist. Each dot is an independent animal. Data are representative of *n* = 3–8 mice per group and are pooled from 3 independent experiments. (**C**) Percentage change in tumor mass in *Batf3*^–/–^ mice treated with CD40 agonist and the indicated cell depletion strategies as compared with *Batf3*^–/–^ mice treated with CD40 agonist only. (**D**) Representative Klrg1 and Lag3 staining of CD8^+^tetramer^+^ T cells from spleens and tumors from untreated *Batf3^+/+^* mice, and *Batf3^+/+^* and *Batf3^–/–^* mice treated with αCD40 on day 14 after tumor. (**E**) Quantification of **D**, gated on CD8^+^tetramer^+^ T cells. (**F**) Representative Foxp3 and Helios staining of CD8^+^tetramer^+^ T cells in spleen and tumor from untreated *Batf3^+/+^* mice and *Batf3^+/+^* and *Batf3^–/–^* mice treated with αCD40 on day 14 posttumor. (**G**) Quantification of **F**, gated on CD8^+^tetramer^+^ T cells. (**H**) Representative Klrg1 and Lag3 staining of CD8^+^tetramer^+^Foxp3^+^ (red) and CD8^+^tetramer^+^ Foxp3^–^ (gray) T cells from spleens of tumor-bearing *Batf3^–/–^* mice treated with αCD40. (**I**) Phenotype of splenic CD8^+^tetramer^+^Foxp3^+^ T cells from tumor-bearing *Batf3^–/–^* mice treated with αCD40 on day 14 posttumor (day 7 after αCD40). (**J**) Functional analysis of Gitr^+^ and Gitr^–^ CD8^+^ T cells following a 5-hour restimulation with CB_101-109_ peptide. *n* = 3–9 mice per group. Data are mean ± SEM and pooled from at least 2 independent experiments. Each dot is an independent animal. One-way ANOVA with a Tukey’s posttest (**B**, **C**, **E**, and **G**). Paired Student’s *t* test (**J**).

To determine the αCD40-mediated *Batf3*-independent antitumor mechanism, we measured tumor weights in *Batf3^–/–^* mice treated with CD40 agonist and the various immune cell depletions used in Figure 8, H and I. We also included additional cohorts in which CD8^+^ T cells were depleted alone or in combination with CD4^+^ T cell depletion. After concatenating samples within cohorts and performing a ViSNE analysis, T cell depletion in both spleen and tumors was robust (Supplemental Figure 8, A and B) and trended to decrease spleen weight (Figure 9A). CD4^+^ and/or CD8^+^ T cell depletion failed to abrogate the benefit of αCD40 in *Batf3^–/–^* mice (Figure 9, B and C). αGr1, which is specific to Ly6G on granulocytes and Ly6C (54), nullified αCD40-mediated antitumor effects (Figure 9, B and C), consistent with an innate antitumor mechanism. Unexpectedly, depletion of monocytes by clodronate liposomes significantly decreased tumor size following αCD40 in *Batf3^–/–^* mice (Figure 9, B and C). These data contrast those of a prior study in the *KPC* model, in which CD40 agonist was shown to be dependent on monocytes (56). To a lesser extent, B cell depletion also enhanced αCD40-mediated antitumor activity (Figure 9, B and C), supporting a protumor role for B cells in this context.

Since T cells primed in the absence of cDC1s failed to participate in tumor control, we hypothesized that priming by monocytes/macrophages alters tumor-specific T cell phenotype and/or function. Therefore, we analyzed tetramer^+^ T cells for the bona fide effector and T_EX_ markers Klrg1, and Lag3, respectively. Indeed, tetramer^+^ T cells primed in *Batf3*^–/–^ mice exhibited an atypical phenotype including an enrichment for a Klrg1^+^Lag3^+^ subset and a significant decrease in a Klrg1^+^Lag3^–^ Teff subset (Figure 9, D and E). Further, tetramer^+^ T cells primed in *Batf3*^–/–^ mice were also enriched for Foxp3 and Helios (Figure 9, F and G). Foxp3^+^tetramer^+^ T cells in *Batf3*^–/–^ mice were distinct from the PD-1^+^Klrg1^+^ subset (Figure 9H). Foxp3^+^tetramer^+^ T cells were CD44^+^, lacked CD40L, and mostly lacked PD-1 and Lag3 (Figure 9I). Most Foxp3^+^tetramer^+^ T cells expressed Gitr (Figure 9I), and we therefore used Gitr as a cell surface surrogate for Foxp3 to quantify intracellular cytokine production following CB_101-109_ stimulation. Gitr^+^CD8^+^ T cells isolated from both spleen and tumor of αCD40-treated *Batf3*^–/–^ mice exhibited higher levels of proinflammatory markers, including IFN-γ and TNF-α (Figure 9J). CB-specific T cells primed in wild-type mice and transferred into tumor-bearing *Batf3^–/–^* mice did not acquire Foxp3 (Supplemental Figure 8C) or the Klrg1^+^Lag3^+^ subset (Figure 7H). Together, our results suggest that the APC that primes tumor-specific T cells affects T cell fate and functionality. As the markers Gitr, Lag3, Helios, and Foxp3 are associated with regulation, priming by monocytes/macrophages appear to produce suboptimal antitumor T cells.

## Discussion

We investigate the role of myeloid subsets on T cell priming, differentiation, and antitumor function in PDA mouse models that permit longitudinal analysis of endogenous tumor-specific T cells (36, 37). Our data converge on a critical role for cDC1s in both priming and reactivation of tumor-specific CD8^+^ T cells and are consistent with other studies (16, 19, 24). Through adoptive T cell transfers and conditional cDC1 depletion, we demonstrate that cDC1s are critical during multiple stages of an antitumor T cell response. Particularly, cDC1s supported a reservoir of splenic Klrg1^+^ cytotoxic T cells, which based on our prior work, are likely necessary for long-term tumor control (36, 37). Analysis of clinical samples supported an antitumor positive feedback circuit between KLRG1^+^ effector T cells and cDC1s. Unexpectedly, we identified that CD40 agonist had partial yet substantial antitumor activity in *Batf3*^–/–^ mice, which lack cDC1s. Further, CD40 agonist promoted the priming of tumor-specific CD8^+^ T cells in *Batf3*^–/–^ mice, which occurs via monocytes/macrophages. However, CD8^+^ T cells primed in cDC1-deficient hosts exhibited a regulatory phenotype and failed to participate in tumor control. As malignancies such as pancreatic and breast cancer interfere with cDC1 development (9, 11), this alternative T cell priming may be more detrimental than beneficial and underscores an essential role for cDC1s in adaptive immunity to carcinomas.

Well-recognized suppressive cells in the pancreatic TME include TAMs, MDSCs, and Tregs (8, 57, 58). High densities of CD15^+^ARG1^+^ granulocytes and TAMs are associated with shortened PDA patient survival (59). In human renal cancer, T_EX_ express Csf-1, thereby promoting TAMs (60). We showed that T_EX_ infiltrating PDA produce IL-10 (37), which may also promote protumor TAMs. Here, TAMs and/or Gr-MDSCs have prominent protumor effects in nAg^–^ and T cell–poor tumors, while appearing dispensable in nAg^+^ and T cell rich tumors. Both phenotype and function of TAMs were altered in nAg^+^ and T cell–rich tumors, suggesting that tumor-specific T cells alter TAM composition. Similarly, transfer of TCR-engineered T cells specific to mesothelin altered TAM composition, and TAM depletion had no benefit on engineered T cell infiltration or functionality in autochthonous PDA (46). Changes in myeloid phenotype following peptide vaccination (61), combination TLR7 agonist with CD200R blockade (62), and IL-12 gene transfer (63) support T cell–mediated modulation of myeloid cells. Thus, suppressive functions of some myeloid subsets may be perturbed when a tumor-specific T cell response is sufficiently engaged and may in part explain the heterogeneity in clinical outcomes following myeloid-targeted therapies (64). Our data suggest that TAM and/or Gr-MDSC ablation may be particularly beneficial for relieving suppression of naive T cell responses to weakly immunogenic antigens. Additionally, TAMs and MDSCs can have protumor roles in a T cell–independent manner, such as by promoting tumor angiogenesis, matrix remodeling, and producing cytokines (65), all of which may be relevant in poorly antigenic tumors.

We show that combination immunotherapy regimens including αPD-1 + αPD-L1 (36) or agonistic αCD40 + αPD-L1 (37) converge on expanding splenic cDC1s. These regimens also expand tumor-specific T cells (36, 37), and Teffs produce several DC-promoting factors, including Xcl1 (21), Flt3L (66), and GM-CSF (67). Thus, while tumor-derived factors can interfere with cDC1 development in PDA (10–14), antigenicity and/or immunotherapy, both of which promote tumor-specific T cells (36, 37), override tumor-dependent cDC1 repression. cDC1 accumulation was most overt in the spleen following immunotherapy, a site we believe is critical for sustaining tumor-specific T cells (36, 37).

Our study highlights the challenges associated with the specific and sustained targeting of cDC1s in vivo. Conditional cDC1 depletion prior to tumor implantation failed to phenocopy tumor burden in *Batf3*^–/–^ mice, highlighting either cDC1-independent defects in *Batf3*^–/–^ mice and/or insufficient cDC1 depletion in the conditional model, particularly when used with immunotherapies that promote cDC1s. *Xcr1*^DTR/DTR^ mice provide an opportunity to study the role of Xcr1 signaling in cDC1s, which we find to promote cDC1 accumulation in tumor-bearing hosts, whereas *Xcr1*^DTR/–^ mice can specifically target cDC1s with at least 1 allele of Xcr1. A recent meta-analysis demonstrated broad expression of *Batf3* in multiple myeloid cells while validating the specificity of Xcr1 as a cDC1-specific marker (28). To circumvent this issue, we performed adoptive transfers of effector or memory *Batf3^+/+^* tumor-specific T cells into tumor-bearing *Batf3^+/+^* or *Batf3*^–/–^ hosts. Our results support that cDC1s are required for the reactivation of tumor-specific memory T cells, resulting in their differentiation into the Klrg1^+^Lag3^–^ effector subset, which appear critical for tumor control (36, 37).

Prior studies suggest *Batf3* is required for antitumor activity when CD40 agonist is used in combination with chemotherapy and immune checkpoint blockade, for the most part in subcutaneous tumor models (32, 68). We show CD40 agonist exhibited partial antitumor activity in *Batf3*^–/–^ mice when CD40 agonist is tested independently in orthotopic tumors with a defined neoepitope. Notably, αPD-L1 + αCD40 significantly prolongs survival and cures approximately 60% of *Batf3*^+/+^ mice (37). Thus, despite some benefit of αCD40 in *Batf3*^–/–^ mice, mice required euthanasia due to achieving a tumor radiance of >10^8^ by day 14, indicating cDC1s are required for long-term durability of αPD-L1 + αCD40 therapy.

*Batf3*^–/–^ mice ultimately reject skin transplants albeit with delayed kinetics compared with *Batf3*^+/+^ mice (69), supportive of a cDC1-independent pathway for T cell–mediated immunity. Here, we find antitumor effects of αCD40 in *Batf3*^–/–^ mice are T cell independent and mediated by the innate immune system. While αGr-1 abrogated the benefit of αCD40, our sustained and high dosing of αGr-1 failed to eliminate Ly6G^+^ cells at endpoint in *Batf3*^–/–^ mice treated with αCD40. Prior studies reveal challenges with sustained granulocyte depletion using αGr-1 (54, 70). Thus, future studies into how αGr-1 impacts granulocytic cell signaling and/or functionality are warranted.

Our investigation of tumor-specific T cells in *Batf3^–/–^* mice unmask unique mechanisms. CD40 agonist primed tumor-specific CD8^+^ T cells independent of cDC1s, which was dependent on monocytes/macrophages. Monocytes require *Irf4* but not *Batf3* to differentiate into *Zbtb46^+^* DCs capable of crosspriming CD8^+^ T cells (71), raising the possibility that agonistic CD40 may promote monocyte differentiation into *Zbtb46^+^* DCs.

In our prior studies, most tumor-specific T cells diverged into Lag3^+^ T_EX_ in PDA or remained Klrg1^+^ functional cytotoxic T cells in the spleens of tumor-bearing mice (36, 37). CD40 agonist promotes the accumulation of the functional intratumoral Klrg1^+^ subset in PDA in wild-type tumor-bearing mice (37). Here, in cDC1-deficient hosts, CD40 agonist primed tumor-specific T cells that coexpressed Klrg1 and Lag3, or a Treg marker Foxp3, and these T cells appeared dispensable for tumor control. *Batf3* has T cell–intrinsic roles in mature CD4^+^ T cells (26, 29) and memory CD8^+^ T cells (27, 53), yet a phenotype has not been identified for CD8^+^ Teffs. Thus, a limitation of our study is we have not ruled out a T cell–intrinsic role for Batf3 in the generation of the atypical T cell subset.

We show that clodronate liposome treatment improved the antitumor response of CD40 agonist in *Batf3^–/–^* mice. Clodronate liposome administration either abrogated the benefit of CD40 agonist in the *KPC* genetically engineered mouse model (56) or promoted antitumor activity of CD40 agonist + gemcitabine in a subcutaneous PDA model in wild-type mice (72). Together, the results support a deeper interrogation of tumor-specific T cell differentiation following CD40 agonist in cDC1-sufficient hosts as our results suggest that competition among cDC1s and macrophages for tumor antigen may shape tumor immunity.

## Methods

### Study design.

We assessed the role of myeloid subsets in preclinical PDA mouse models that differ in antigenicity and T cell infiltration. We pursued this line of investigation by depleting specific myeloid cell subsets and using peptide-MHC tetramers to analyze tumor antigen-specific T cell responses at baseline and following immunotherapy. Through adoptive transfer studies, and cDC1-deficient mouse models, we identify cDC1-dependent and -independent antitumor mechanisms governing immunity to PDA.

### Animals.

The University of Minnesota IACUC approved all animal studies. We backcrossed the *Kras*^LSL-G12D+^ *Trp53*^LSL-R172H/+^ *p48*^Cre^ (*KPC*) mice to >99.6% C57BL/6J background previously (33) to generate the syngeneic *KPC* cell lines for this study. Six- to 12-week-old female and male C57BL/6J (Jackson Laboratory, 000664), *Batf3*^–/–^ (Jackson Laboratory, 013755) (19), and MaFIA mice (Jackson Laboratory, 005070) were used for orthotopic tumor implantation experiments. *Xcr1*^DTRVenus^ mice (30) were a gift from Wakayama Medical University and obtained from Matthew Krummel (UCSF, San Francisco, California, USA) via the University of Minnesota. C57BL/6J Thy1.1 mice (Jackson Laboratory, 000406) were vaccinated for T cell adoptive transfer studies.

### Primary tumor epithelial cells.

We previously described C57BL/6J primary *KPC* tumor epithelial cell lines with or without CB (36). Tumor cells were maintained below passage 15 in basic media—500 mL DMEM (Gibco) +10% FBS (Gibco) + 2.5 μg/mL Amphotericin B (Gibco) + 100 μg/mL pen/strep (Gibco) + 2.5 mg dextrose (Fisher Chemical)—at 37°C and 5% CO_2_ as described (33). Media were sterile filtered and stored at 4°C.

### Orthotopic tumor cell implantation.

After mice reached surgical plane anesthesia, a small incision was made in the right abdomen to access the pancreas. A total of 10,000 *KPC* CB^–^ or CB^+^ were injected into the pancreas in 20 μL of 60% Matrigel (Discovery Labware) using an insulin syringe (Covidien) as described (36). Separate sets of sutures were used to close the peritoneum and skin (Ethicon). Euthanasia criteria for excessive tumor burden are >10^8^ radiance (if tumor cell lines express the CB) or >500 mm^3^, as determined by high-resolution ultrasound (Vevo2100), for parental CB^–^ lines.

### Peptide vaccination.

*Batf3^–/–^* or *Batf3^+/+^* mice were vaccinated intraperitoneally (i.p.) with 100 μg CB_101-109_ peptide along with 50 μg agonistic αCD40 (FGK45, BioXcell) and 50 g Poly I:C (Sigma) (36). At 7 days after vaccination, spleens were dissociated to single cells and for flow cytometric analysis.

### In vivo monoclonal immunotherapy treatments.

Mice received a single dose of 100 μg of agonistic αCD40 (FGK45, BioXcell) i.p. on day 7 alone, or in combination with 200 μg of αPD-L1 (10F.9G2, BioXcell) i.p. on days 7, 10, and 12 after orthotopic tumor implantation as described (37). Mice received 200 μg of αPD-1 (RMP1-14, BioXcell) and 200 μg αPD-L1 (10F.9G2, BioXcell) i.p. on days 7, 10, 12 as described (36).

### In vivo immune cell depletions in Batf3^–/–^ mice.

To deplete CD4^+^ or CD8^+^ T cells, *Batf3^–/–^* mice bearing CB^+^ orthotopic tumors were treated with 200 μg of αCD4 (GK1.5, BioXcell) i.p. on days –1, 1, 4, and/or 200 μg of αCD8 (2.43, BioXcell) i.p. on days –1, 5, and 10. A total of 400 μg of αCD20 (SA271G2, BioLegend) on day –1 relative to tumor implantation was used to deplete B cells. A total of 400 μg of αCSF1R (AFS98, BioXcell), 200 μg of αGr-1 (RB6-8C5, BioXcell), or 200–250 μL of control liposomes or clodronate-loaded liposomes (Encapsula NanoSciences) were administered i.p. on days –1, 1, 3, 5, 7, 9, and 11 relative to orthotopic tumor implantation.

### cDC1 and TAM depletion.

To deplete cDC1 cells, *Xcr1*^DTR/DTR^ mice were treated with 500 ng DT (Sigma) i.p. diluted in sterile saline on days –1, 5, and 10 (DT-Pre) or days 6, 9, and 12 (DT-Post) relative to orthotopic tumor cell implantation. To deplete Csf1r^+^ cells, MaFIA mice (Jackson Laboratory, 005070) were treated with the FK506-dimerizing agent AP20187 (ApexBio) as described (47). Briefly, lyophilized AP20187 was dissolved in 100% ethanol at a concentration of 62.5 mg/mL stock solution and stored at −20°C. Injection solutions consist of 4% ethanol, 10% PEG-400 (MilliporeSigma), and 2% Tween 20 (MilliporeSigma) in water. Mice receiving only the vehicle (4% ethanol, 10% PEG-400, 2% Tween 20 in water) served as controls. MaFIA mice were injected i.p. with 5 mg of AP20187 on days 6, 9, and 12 following orthotopic tumor implantation.

### In vivo imaging.

Abdominal hair was removed with Nair. For bioluminescence imaging, tumor-bearing mice were injected with 100 μg of d-Luciferin (Promega) i.p., and images were acquired after 11 minutes as described (36, 37). Images were first acquired after 0.5-second exposure time with a binning of 8 seconds. As luminescence saturation may occur, additional images with a binning of 2 seconds and/or autoexposure setting were acquired. Tumor radiance was quantified in photons per second using IVIS 100 and Living Image software (Xenogen).

### Production of CB_101-109_:H-2D^b^ fluorescently labeled tetramer.

H-2D^b^-restricted biotinylated monomer was produced by incubating CB_101-109_ peptide (GenScript) and purified H-2D^b^ and β2m (gifts from Masopust lab, University of Minnesota) followed by purification via Fast Protein Liquid Chromatography system (ÄKTAprime plus, GE Healthcare, now Cytiva). Biotinylated monomer was conjugated to streptavidin R-phycoerythrin or streptavidin BV421 (Invitrogen) to produce fluorescent CB_101-109_:H-2D^b^ tetramer (36, 37).

### Preparation of mononuclear cells from tissues.

Spleens were mechanically dissociated to single cells, and RBCs were lysed by incubation in 1 mL of Tris-ammonium chloride (Ack) lysis buffer (Gibco) for 2 minutes at room temperature in 15 mL conical tubes. Next, 9 mL of T cell media—DMEM (Gibco) + 10% FBS (Gibco), 100 μg/mL pen/strep (Gibco), 20 mM l-glutamine (Gibco), 1× nonessential amino acids (Gibco), and 50 μM β-mercaptoethanol (Sigma)—was added to quench lysis. Cells were centrifuged at 300*g* for 5 minutes and stored in T cell media on ice. Tumors were mechanically digested to single cells in a similar manner including 2 additional wash steps to remove cell debris and pancreatic enzymes.

### Flow cytometric analyses of mononuclear cells from tissues.

To profile the phenotype of splenic and intratumoral T cells, mononuclear cells were stained with CB_101-109_:H-2D^b^-PE or -BV421 tetramer (1:100) in the presence of 1:500 Fc block (CD16/32, Tonbo) along with monoclonal antibodies against the following cell surface markers diluted at 1:200 in FACS buffer (PBS+2.5% FBS): CD45 (clone 30F-11, BioLegend), CD8 (clone 53-6.7, Tonbo), CD3 (clone 17A2, BioLegend), CD4 (clone GK1.5, BD Biosciences), CD44 (clone IM7, BD Biosciences), PD-1 (clone J43, Invitrogen), Lag3 (clone C9B7W, BioLegend), Klrg1 (clone 2F1, BioLegend), Tim3 (clone RMT3-23, BioLegend), TIGIT (clone 1G9, BD Biosciences), and GITR (clone DTA-1, eBioscience). To determine myeloid cell composition, single-cell suspensions were stained with 1:500 Fc blockade (CD16/32, Tonbo) and Tonbo Ghost dye in BV510 (1:400) along with antibodies (1:200 in FACS buffer) specific to CD45 (clone 30F11, BioLegend), CD3 (clone 17A2, BioLegend), CD19 (clone 1D3, BD Biosciences), NK1.1 (clone PK136, eBioscience), CD11b (clone M1/70, Tonbo), Ly6G (clone 1A8, eBioscience), CD64 (clone X54-5/7.1, BioLegend), F4/80 (clone BM8, eBioscience), CD11c (clone N418, BD Biosciences), I-A/I-E (clone M5/114.15.2, BioLegend), CD8α (clone 53-6.7, Tonbo), CD103 (clone M290, BD Biosciences), Xcr1 (clone ZET, BioLegend), SIRPα (clone P84, BioLegend), CD115 (clone AFS98, eBioscience), PD-L1 (clone 10F.9G2, BioLegend), and/or CD40 (clone HM40-3, BioLegend) for 30 minutes at 4°C in the dark. Cells were fixed with 0.4% paraformaldehyde for 15 minutes and analyzed immediately or stored at 4°C in the dark and acquired the next day using a Fortessa 1770 and FACSDiva software (BD Biosciences). FlowJo software (version 10) was used for data analysis. For Supplemental Figure 8A, Cytobank software was used for ViSNE analysis.

### Intracellular cytokine production.

Mononuclear cell suspensions of spleen and tumor were cultured in complete T cell media in the presence of Golgiplug (1:500 BD Biosciences) and CB_101-109_ peptide (1 μg/mL GenScript) for 4–5 hours at 37°C, 5% CO_2_. Next, cells were stained for Ghost live-dead (Tonbo) at 1:400 along with cell surface antibodies at 1:200 diluted in FACS buffer including CD45 (clone 30F-11, BioLegend), CD8α (clone 53-6.7, Tonbo), CD44 (clone IM7, BD), and Klrg1 (clone 2F1, BioLegend). Cells were then fixed and permeabilized (BD Fixation Kit) and stained for intracellular expression of IFN-γ (XMG1.2, BioLegend) and TNF-α (MP6-Xt22, BioLegend) at 1:100 dilution overnight in the dark at 4°C per manufacturer’s instructions.

### Intracellular Foxp3, Helios, Arg1, and iNOS staining.

Single-cell suspensions of splenic or intratumoral cells were stained for 30 minutes at 4°C in the dark with Ghost live-dead (Tonbo, 1:400) and cell surface antibodies CD45 (clone 30F-11, BioLegend), CD4 (clone GK1.5, BD), CD44 (clone IM7, BD), CD8, and dump (CD19, F4/80) diluted 1:200 in FACS buffer. Cells were washed with FACs buffer twice, fixed, and permeabilized using Foxp3 staining kit (Tonbo) followed by intracellular staining with antibodies against Foxp3 (clone FJK-16s, eBioscience) and Helios (clone 22F6, BioLegend) diluted 1:100 in perm/wash buffer for 2 hours at 4°C at 1:100 dilution per manufacturer’s instructions. Cells were washed twice with perm/wash buffer, stored at 4°C, and acquired within 24 hours following staining. For Figure 1A, cells were fixed and permeabilized (BD Fixation Kit), stained for intracellular expression of Arg1 (clone A1exF5, eBioscience) and iNOS (clone CXNFT, eBioscience) at 1:100 dilution in perm/wash buffer for 2 hours at 4°C in the dark, and acquired within 24 hours following staining.

### Immunofluorescence.

Tissues were embedded in OCT (Tissue-Tek) and stored at –80°C. Sections of 7 μm were cut using a cryostat and fixed in acetone at –20°C for 10 minutes. Sections were rehydrated with PBS + 1% BSA and incubated for 1 hour at room temperature with various combinations of primary antibodies PD-L1 (clone AF1019, R&D Systems, 1:500), CD8α (BD Biosciences, clone 53-6.7, 1:100), pan-cytokeratin–FITC (clone F3418, MilliporeSigma, 1:200), F4/80 (clone BM8.1, Tonbo, 1:100), and Ly6G (clone 1A8, BD, 1:100) diluted in PBS + 1% BSA. Slides were washed 3 times in PBS + 1% BSA and incubated with anti-goat AF647 (Jackson ImmunoResearch, 1:500) for 1 hour at room temperature in the dark. Following staining, slides were then washed 3 times with PBS + 1% BSA, washed 3 times with PBS, and mounted in DAPI Prolong Gold (Life Technologies). Images were acquired on a Leica DM6000 epifluorescence microscope at the University of Minnesota Center for Immunology using Leica LasX software and analyzed using Fiji2.0.

### Calculation of cell numbers normalized to tissue gram.

Flow cytometry cell counting beads (Thermo Fisher Scientific) were added to each sample. The number of live CD45^+^ cells collected per tube was determined using FlowJo analysis software and the following equation: #CD45^+^ cells per tube (*n*) = (#beads/#cells) × (concentration of beads × volume of beads added). Total number of cells collected from the entire single-cell suspension was determined by multiplying *n* by total number of stains. All cell number data presented in this study are normalized to tissue gram. This was accomplished by dividing cell number by tissue weight in grams that was used to generate the single-cell suspension for flow cytometry.

### Adoptive transfer of CB_101-109_-specific Thy1.1^+^ effector or memory CD8^+^ T cells.

Thy1.1^+/+^ mice were vaccinated with αCD40+CB_101-109_+Poly(I:C) as described above and previously (36). Teffs were harvested 7 days postvaccination from spleen, pooled, and transferred to *Batf3^+/+^* or *Batf3^–/–^* mice at day 3 following orthotopic *KPC*2a tumor cell implantation. Recipient mice received 14,000 Thy1.1^+/+^CD8^+^ CB_101-109_-specific Teffs. Spleens and tumors were harvested from Teff recipients 7 days after T cell transfer, e.g., 10 days posttumor. Thy1.1^+/+^ splenocytes containing memory CD8^+^ T cells from separate cohorts of vaccinated mice were harvested on day 41 after vaccination. A total of 4000 Thy1.1^+/+^CD8^+^ CB_101-109_-specific memory T cells were transferred into recipient *Batf3^+/+^* or *Batf3^–/–^* mice immediately prior to tumor implantation. Spleen and tumor from memory T cell recipients were harvested 14 days after T cell transfer for analysis.

### Statistics.

Statistical analyses were performed using GraphPad software (version 7.0 or above). All mouse experiments reflect *n* = 3–8 mice per group. Student’s 2-tailed *t* test was used to compare 2-group data. One-way ANOVA and Tukey’s posttest were used for comparison of >2-group data. Log-rank (Mantel-Cox) test was used to test for statistically significant differences in mouse survival. Data are presented as mean ± SEM and *P* < 0.05 was considered significant. *, *P* < 0.05; **P < 0.005; ***, *P* < 0.0005.

### Study approval.

The mouse studies were approved by the IACUC at the University of Minnesota.

## Author contributions

ALB and IMS conceptualized the study, analyzed the data, and wrote the manuscript. ZCS and EJS edited the manuscript. ALB, ZCS, MTP, and EJS conducted experiments. EAM, MRR, and JFR assisted with experiments. JSM, TK, and BTF provided *Xcr1*^DTR/DTR^ mice. All authors reviewed the manuscript.

## Supplementary Material

Supplemental data

## Version 1 04/08/2022

Electronic publication
