# Supplementary material for: Distinct myeloid antigen-presenting cells dictate differential fates of tumor-specific CD8+ T cells in pancreatic cancer
Source: JCI Insight. 2022 Apr 8;7(7):e151593. doi: 10.1172/jci.insight.151593 (PMC9057584; doi:10.1172/jci.insight.151593)
Supplement: Supplemental data [file jciinsight-7-151593-s076.pdf]

Supplementary Figure 1

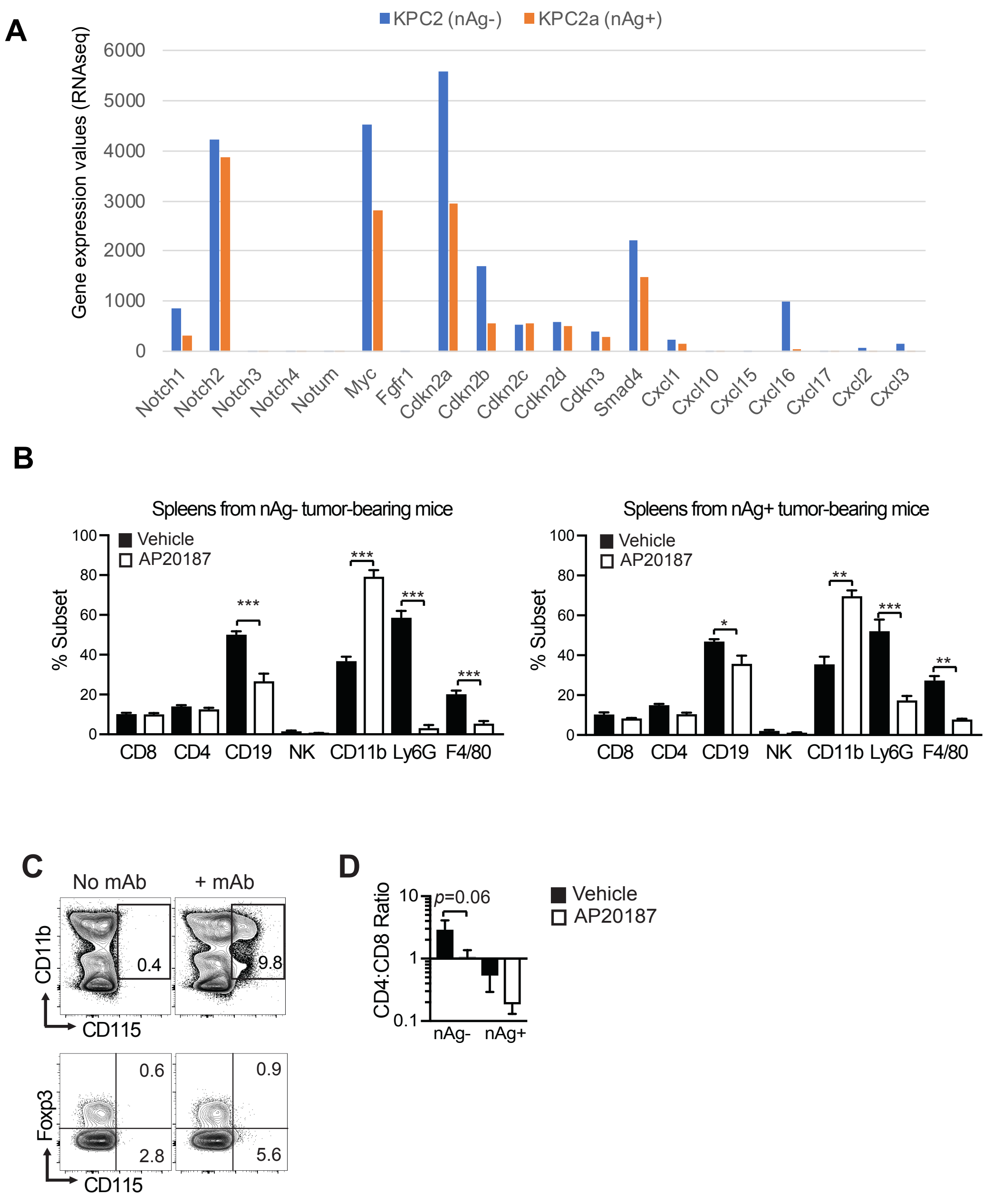

Supplementary Figure 1. Role of tumor antigenicity on myeloid cells in pancreatic cancer. A) Gene expression of CB+ and parental CB- *KPC* tumor cell line was determined by RNAseq. **B**) Mean frequency of immune cell subsets in the spleens from orthotopic tumor-bearing mice on day 14 posttumor. *p<0.05, **p<0.005, and ***p<0.0005 (student's T test). **C**) Representative staining of CD115 (Csf1r) on positive control (top, CD11b+ myeloid cells) or CD4+T cells (bottom). **D**) Ratio of CD4:CD8 T cells in nAg- or nAg+ tumors on day 15. Student's T test to compare ratio of 2 group data within each tumor cohort.

Supplementary Figure 2

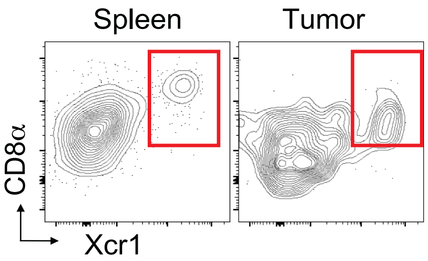

Supplementary Figure 2. cDC1s co-express CD8α and Xcr1 in orthotopic pancreatic tumor-bearing mice. Representative FACs plots gated on live CD45+Lin-CD11c+MHCII+ cells on day 14 posttumor. Representative of n=5 mice per group.

Supplementary Figure 3

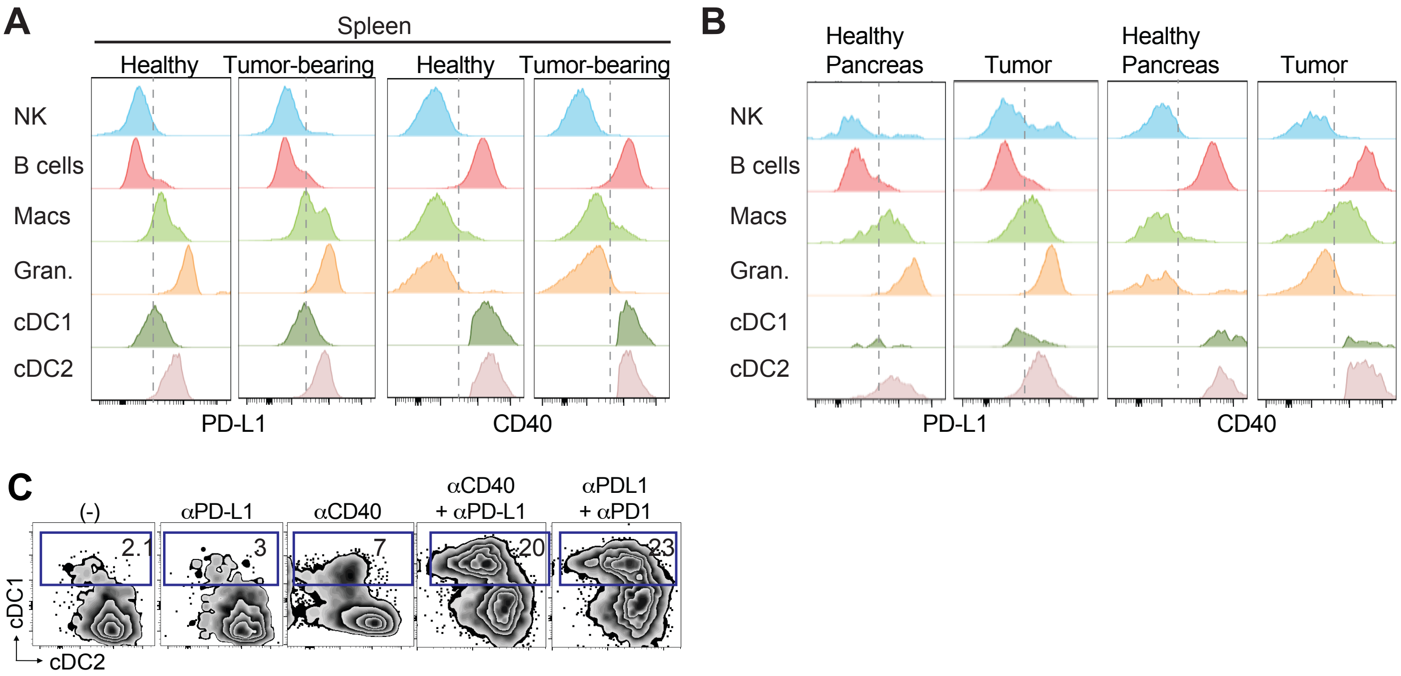

Supplementary Figure 3. Expression of PD-L1 and CD40 by immune cells in nAg+ tumor-bearing mice. **A**) Representative histograms of PD-L1 and CD40 expressed by the indicated splenic cell subsets from healthy or nAg+ tumor-bearing mice on day 21 posttumor. **B**) Representative histograms of PD-L1 and CD40 expressed by immune cells isolated from healthy pancreas or orthotopic nAg+ tumors on day 21 posttumor. **C**) Representative flow plots gated on live CD45+CD3-CD19-NK1.1-F4/80-Ly6G-CD11c+MHC class II+ cells from spleen on day 14 post tumor implantation (day 7 post immunotherapy).

Supplementary Figure 4

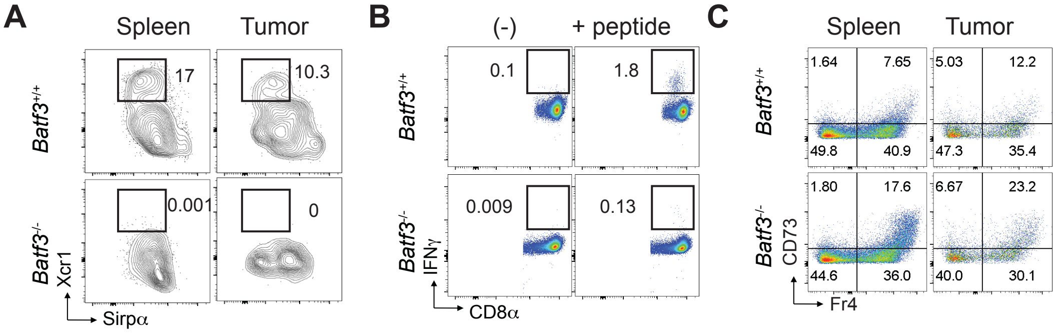

Supplementary Figure 4. Failure to generate tumor-specific T cells and altered CD4 T cell phenotype in *Batf3*$^{-/-}$ mice. **A**) Undetectable cDC1s in *Batf3*$^{-/-}$ tumor-bearing mice. Representative FACs plots of cDC1s (box) gated on live, CD45+Lin-CD11c+MHCII+ cells. n=5 mice per group. **B**) Undetectable IFNγ+ T cells in *Batf3*$^{-/-}$ tumor-bearing mice. Representative staining of splenocytes restimulated with $CB_{101\text{-}109}$ peptide for 4 h *ex vivo* with Golgiplug, stained for intracellular IFNγ and analyzed by flow cytometry. Representative of n=4 mice per group. **C**) Expression of anergic T cell markers CD73 and Fr4 in conventional CD4+Foxp3- T cells isolated from spleen or tumor from *Batf3*$^{+/+}$ or *Batf3*$^{-/-}$ mice on day 14 posttumor. Representative of n=5 mice per group.

Supplementary Figure 5

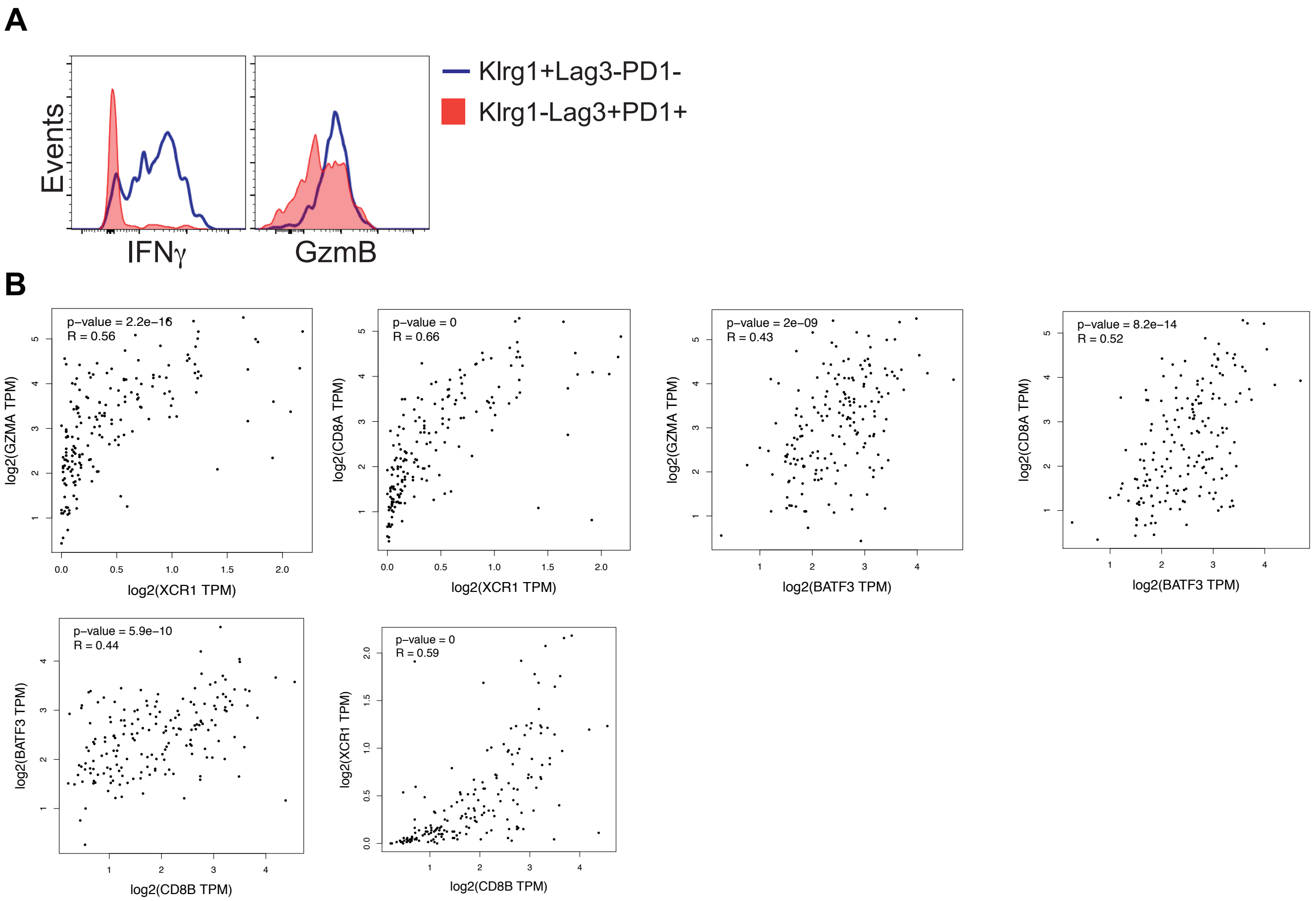

Supplementary Figure 5. Klrg1 and Lag3 are surrogate markers for two functional states of T cells in PDA and cytotoxic T cell genes correlate with cDC1 genes in human PDA. A) IFNγ and Granzyme B (GzmB) production from intratumoral Klrg1^{+}Lag3^{-}PD1^{-} vs. Klrg1^{-}Lag3^{+}PD1^{+} CD8^{+} T cells cells following a 5 h *in vitro* stimulation with PMA and Ionomycin + Golgiplug on day 14 posttumor was determined by flow cytometry. **B)** Correlation between the cDC1 genes *XCR1* and *BATF3* and CD8 T cell genes from 178 human PDA samples using TCGA dataset and GEPIA (http://gepia.cancer-pku.cn/).

Supplementary Figure 6

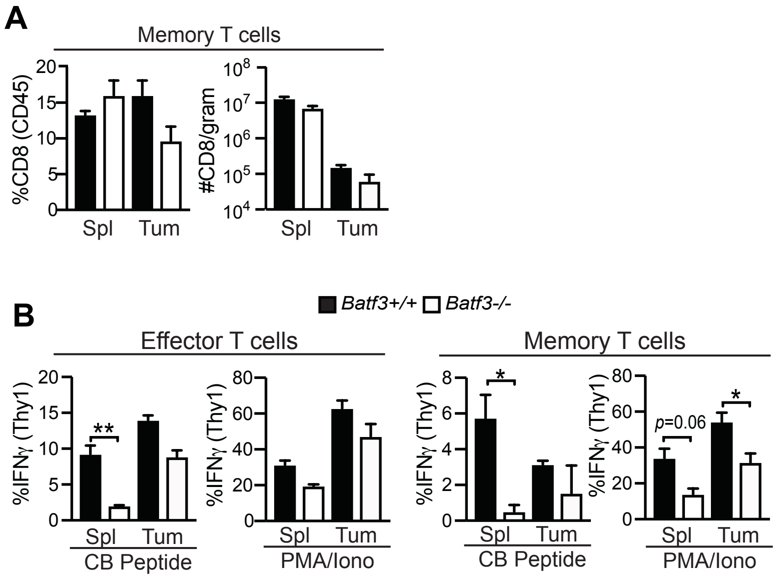

Supplementary Figure 6. Host cDC1s are critical for re-activating adoptively transferred effector memory T cells. A) Proportion of total CD8 T cells and overall number normalized to tissue gram in *Batf3*$^{+/+}$ and *Batf3*$^{-/-}$ tumor-bearing recipients of memory T cells. **B)** Proportion of Thy1.1+ (Thy1) T cells that produce IFNγ in response to specific antigen (peptide) or non-specific stimulation (PMA+Ionomycin). Data are mean ± S.E.M. n=5 mice per group. *p<0.05. Student's t-test was used to compare 2-group data.

Supplementary Figure 7

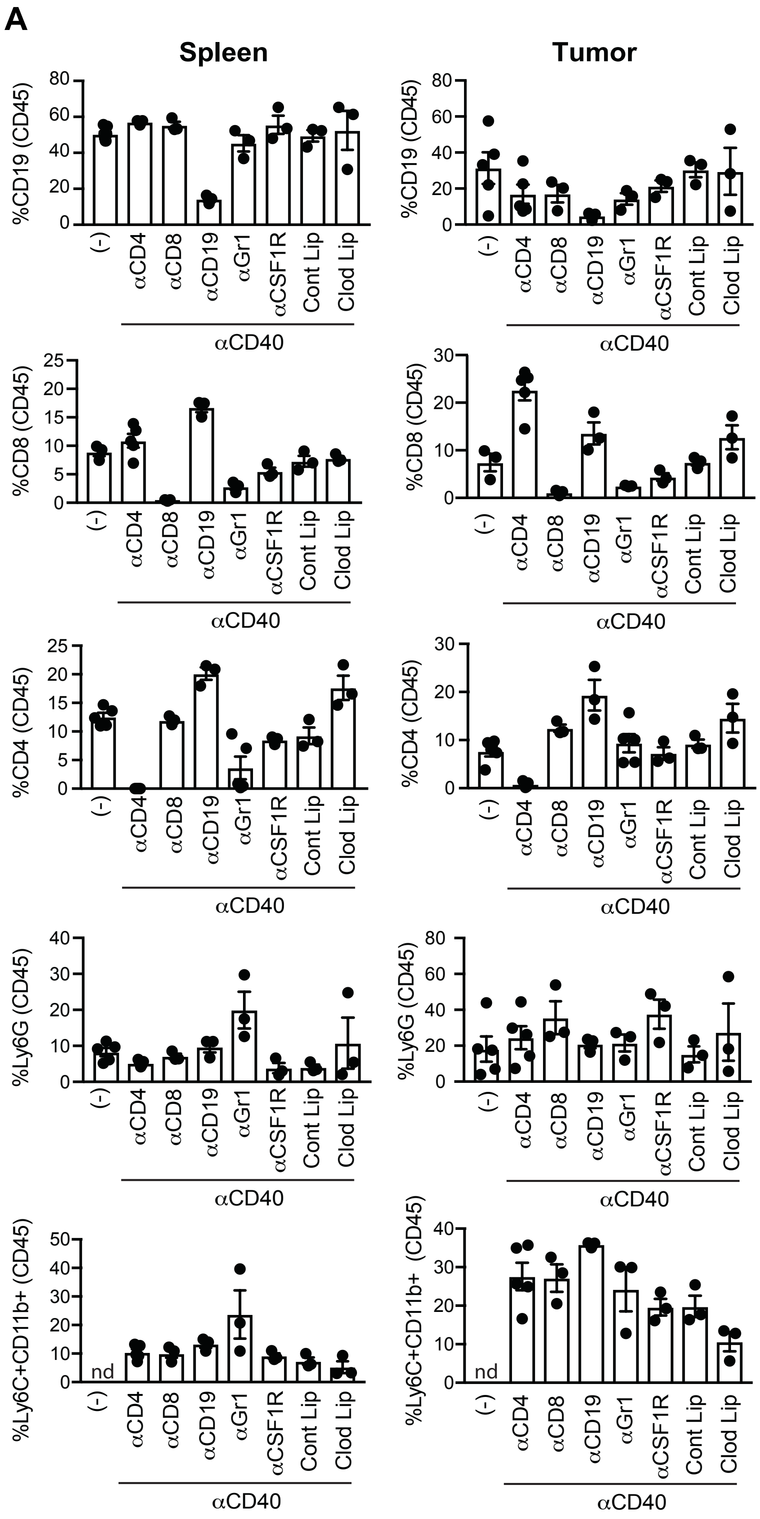

Supplementary Fig. 7. CD40 agonist primes tumor-specific CD8 T cells independent of Batf3. A) Quantification of the indicated cell types that were targeted for depletion in *Batf3*$^{-/-}$ mice prior to αCD40. Each dot is an independent mouse. Data are mean ± S.E.M and pooled from up to 3 independent experiments, depending on the regimen.

Supplementary Figure 8

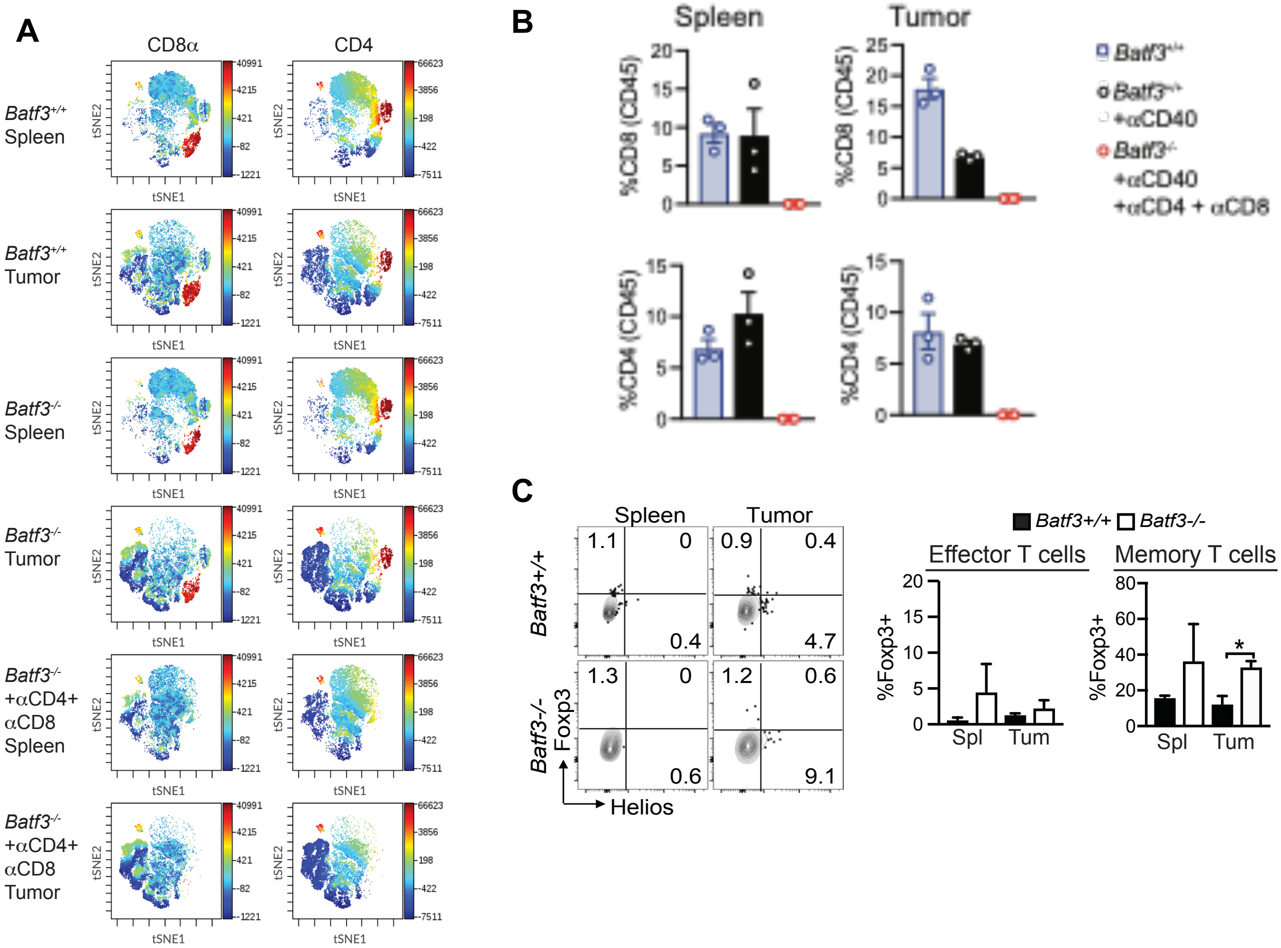

Supplementary Figure 8. Antitumor activity in *Batf3*$^{-/-}$ mice treated with CD40 agonist is T cell independent. A) Visne analysis of concatenated flow cytometry samples from each cohort were analyzed using Cytobank software. **B**) Quantification of the frequency of CD4 and CD8 T cells among total CD45+ cells in mice from A. Data are mean ± SEM. n=3-4 mice per group. **C**) Phenotypic analysis of donor Thy1.1+CD8+tetramer+ T cells (see Figure 7) in *Batf3*$^{+/+}$ or *Batf3*$^{-/-}$ tumor-bearing mice. Graphed data are mean ± S.E.M. *, $p<0.05$, student's T test to compare 2 group data (spleen or tumor assessed).
